# Beyond confidence: Development of a measure assessing the 5C psychological antecedents of vaccination

**DOI:** 10.1371/journal.pone.0208601

**Published:** 2018-12-07

**Authors:** Cornelia Betsch, Philipp Schmid, Dorothee Heinemeier, Lars Korn, Cindy Holtmann, Robert Böhm

**Affiliations:** 1 CEREB - Center for Empirical Research in Economics and Behavioral Sciences, University of Erfurt, Erfurt, Germany; 2 Media and Communication Science, University of Erfurt, Erfurt, Germany; 3 School of Business and Economics, RWTH Aachen University, Aachen, Germany; University of Campania, ITALY

## Abstract

**Background:**

Monitoring the reasons why a considerable number of people do not receive recommended vaccinations allows identification of important trends over time, and designing and evaluating strategies to address vaccine hesitancy and increase vaccine uptake. Existing validated measures assessing vaccine hesitancy focus primarily on confidence in vaccines and the system that delivers them. However, empirical and theoretical work has stated that complacency (not perceiving diseases as high risk), constraints (structural and psychological barriers), calculation (engagement in extensive information searching), and aspects pertaining to collective responsibility (willingness to protect others) also play a role in explaining vaccination behavior. The objective was therefore to develop a validated measure of these 5C psychological antecedents of vaccination.

**Methods and findings:**

Three cross-sectional studies were conducted. Study 1 uses factor analysis to develop an initial scale and assesses the sub-scales’ convergent, discriminant, and concurrent validity (*N* = 1,445, two German convenience-samples). In Study 2, a sample representative regarding age and gender for the German population (*N* = 1,003) completed the measure for vaccination in general and for specific vaccinations to assess the potential need for a vaccine-specific wording of items. Study 3 compared the novel scale’s performance with six existing measures of vaccine hesitancy (*N* = 350, US convenience-sample). As an outcome, a long (15-item) and short (5-item) 5C scale were developed as reliable and valid indicators of confidence, complacency, constraints, calculation, and collective responsibility. The 5C sub-scales correlated with relevant psychological concepts, such as attitude (confidence), perceived personal health status and invulnerability (complacency), self-control (constraints), preference for deliberation (calculation), and communal orientation (collective responsibility), among others. The new scale provided similar results when formulated in a general vs. vaccine-specific way (Study 2). In a comparison of seven measures the 5C scale was constantly among the scales that explained the highest amounts of variance in analyses predicting single vaccinations (between 20% and 40%; Study 3). The present studies are limited to the concurrent validity of the scales.

**Conclusions:**

The 5C scale provides a novel tool to monitor psychological antecedents of vaccination and facilitates diagnosis, intervention design and evaluation. Its short version is suitable for field settings and regular global monitoring of relevant antecedents of vaccination.

## Introduction

According to estimates by the World Health Organization (WHO), vaccination saves 2 to 3 million lives worldwide every year [1]. Nevertheless, a considerable number of children or adults are not getting vaccinated [2], leading to outbreaks of vaccine-preventable diseases and avoidable deaths, such as from measles or pertussis. *Vaccine hesitancy*, as it is frequently termed, is a global phenomenon [3,4] and national as well as international health organizations have partnered with academia to understand its causes and curtail its consequences [5,6].

There is broad consensus that a valid and reliable measure to diagnose why people do not vaccinate will support the design and evaluation of interventions that aim to increase vaccine uptake [6–8]. Such an agreed-upon measure is also needed as WHO asks countries to monitor and report vaccine hesitancy in their annual joint reporting form (JRF) to monitor changes and trends over time and to detect vaccine concerns early [9]. In 2016, only about one third of countries reported based on the evaluation of actual data, demonstrating the need for a simple but valid measure [10]. Standardized measures will also make future research results easier to compare (e.g., in meta-analyses), improve data quality over time, and facilitate the development of evidence-based interventions [11].

In the present contribution, we devise a novel measure to capture relevant predictors of vaccination behavior–the 5C scale, which measures the “psychological antecedents of vaccination” as suggested in a recent review by Brewer and colleagues [12]. The 5C scale is grounded in established theoretical models of vaccine hesitancy and acceptance [5,13,14] and relates these to psychological models to explain health behavior [7]. We conducted three studies with nearly 2,800 participants testing its convergent, discriminant, and concurrent validity, resulting in a reliable and valid long (15-item) and short (5-item) version of the scale. Before describing the scale’s development process in more detail, the following sections provide an overview of several models explaining vaccination behavior and existing measures to assess its determinants. Based on these elaborations, we will justify the need for the newly developed 5C scale as another building block offered in the collective endeavor of globally improving vaccine uptake.

### Vaccine hesitancy, confidence, and acceptance: Models to understand vaccination behavior

There is some debate in the literature how we should name the fact that some people do not vaccinate–hesitancy, a lack of confidence or trust, and low acceptance are often used interchangeably [6,12]. The first international systematic endeavor was a working group of a WHO advisory body (Strategic Advisory Group of Experts on Immunization, SAGE), defining *vaccine hesitancy* as the “delay in acceptance or refusal of vaccines despite availability of vaccine services. Vaccine hesitancy is complex and context specific, varying across time, place and vaccines. It is influenced by factors such as complacency, convenience and confidence” [15]. Hesitancy thus describes a continuum between complete acceptance and complete refusal [5]. The factors complacency (not perceiving diseases as high risk and vaccination as necessary), convenience (practical barriers) and confidence (lack of trust in safety and effectiveness of vaccines)–referred to as the 3C model–were identified based on experience in various countries [16] and extensive literature review [13]. Table 1 provides their exact definitions (column 1).

**Table 1:** Overview of models of explaining vaccine hesitancy, confidence or acceptance and corresponding general measures.

Models			Measures						
SAGE Working group: The 3C model [5]	The 4C model (extended 3C model) [7]	The 5A model: Taxonomy for the determinants of vaccine uptake [14]	Parental Attitudes about Childhood Vaccines (PACV, Opel) [17]	Vaccine Confidence Scale (VCS, Gilkey) [18]	Global Vaccine Confidence Index (GVCI, Larson) [4]	Vaccine Hesitancy Scale (VHS, Shapiro) [19]	Vaccine Acceptance Scale (VAS, Sarath-chandra) [20]	Vaccine Confidence Index (VCI, Frew) [21]	5C antecedents of vaccine acceptance (5C)
3 factors	4 factors	5 determinants	15 items 3 sub-scales	8 items 3 sub-scales	4 items no sub-scales	9 items 2 sub-scales	20 items 5 sub-scales	8 items 3 sub-scales	5 or 15 items 5 sub-scales
confidence: trust in effectiveness and safety of vaccines and the system that delivers them (health care workers, politics)	confidence: negative attitudes, belief in misinformation, perceptions of vaccine-related risks	acceptance: individuals accept, question or refuse vaccination	beliefs about safety and efficacy general attitudes and trust immunization behavior	benefits trust harms	safety effectiveness	lack of confidence risks of vaccination	perceived safety of vaccines perceived effectiveness and necessity of vaccines acceptance of the selection and scheduling of vaccines positive values and affect toward vaccines perceived legitimacy of authorities to require vaccinations	trust confidence importance	confidence
convenience: physical availability, affordability and willingness-to-pay, geographical accessibility, ability to understand (language and health literacy) and appeal of immunization service	convenience: structural barriers, perceived behavioral control	access: ability of individuals to be reached by, or to reach, recommended vaccines affordability: ability of individuals to afford vaccination	--	--	--	--	--	--	Constraints
complacency: perceived risks of diseases are low; vaccination not seen as necessary	complacency: low involvement, low general knowledge, awareness, vaccination not seen as the injunctive norm	awareness: knowledge (need for/availability of vaccines)	--	--	importance	--	--	--	Complacency
--	calculation: individuals’ engagement in extensive information search		--	--	--	--	--	--	Calculation
**--**	**--**	activation: degree to which individuals are nudged towards vaccination uptake	--	--	--	--	--	--	--
		(awareness: social benefits)	--	--	--	--	--	--	collective responsibility
			--	--	compatibility with religious beliefs	--	--	--	--

The left part of this table shows that the approaches to understanding vaccine hesitancy and acceptance differ in the number of concepts and the level of specificity with which the concepts are defined. The right part provides an overview of existing measures and relates the factors assessed by the measures to the theoretical models. The 5C scale assesses all relevant concepts as documented in the literature at the most fine-graded level of specificity (last column). Categorizing of the sub-scales is based on the authors’ assumptions and has been cross-validated in personal communication with the original scales’ authors for PACV, VCS, and VHS.

The definitions show that these factors comprise several concepts from psychological theories for predicting prevention behavior [22]. For example, confidence includes behavioral beliefs about vaccination (knowledge), which relate to the attitude towards vaccination [23]. In previous work [22], we therefore suggested constellations of psychological predictors that match the hesitancy factors’ definitions (given in Table 1, column 2), referring to established theoretical frameworks, such as the Health Belief Model [24] and the Theory of Planned Behavior [23]. Based on literature reviews and theoretical considerations, we extended the 3C model to a 4C model (column 2) by integrating calculation (the individual’s engagement in extensive information searching) as an additional psychological antecedent. This factor captures the individual motivation of thinking about and questioning vaccination [22] and is often positively correlated with vaccine hesitancy [9,25].

The 5A taxonomy for explaining vaccine uptake provides a somewhat different terminology. It is based on a narrative review and identifies five categories labeled acceptance, access, affordability, awareness, and activation (definitions in Table 1, column 3) [14]. As is obvious from Table 1, there is considerable overlap to the categories proposed by the SAGE working group: acceptance resembles the confidence factor (attitude as a potential linkage), access matches convenience (practical barriers), likewise affordability (financial costs; practical and financial barriers are included in the convenience factor), and awareness is comparable to complacency (lack of the perception that the diseases are high risk and that vaccination is necessary). The 5A taxonomy adds an additional aspect–activation–which notes the existence of nudges in the environment that may increase vaccination (e.g., reminders [14]). As we intend to measure individual psychological differences, we suggest assessing the individual proneness to nudges (system 1 processing) vs. a need for thorough information processing (system 2) [26]. The concept of calculation thus expresses the need of extensive elaboration and information searching. Finally, the awareness concept also includes the social benefits of vaccination, i.e., the fact that due to herd immunity most vaccinations also protect unvaccinated individuals [27]. This concept resonates in the fifth factor of the 5C scale–collective responsibility–which was added as a result of Study 1.

The term hesitancy has been criticized as its behavioral definition neglects that hesitancy can also be a psychological state of indecision and that any type of non-vaccination is now labeled hesitancy, even though access, system failures or total refusal may be the cause [28]. Further, the term convenience puts the responsibility of receiving a vaccine to the individual, mixing up social determinants with access. Convenience suggests low parental prioritization, however, the reason may be rooted in the system that delivers the vaccines [28]. The 5A model circumvents this by using “access” as a separate construct. In a similar vein, we propose “constraints” as a synonym for perceived barriers as a new term for convenience.

As there is no agreed upon definition of the phenomenon of “vaccine hesitancy”, in this work we avoid “hesitancy” as a conceptual umbrella for the 5C scale. Additionally, lacking a definition of hesitancy makes it difficult to establish external criteria to assess content validity. Thus, we decided to construct a scale that assesses psychological antecedents of vaccination and offer correlations with psychological constructs to estimate content validity of each sub-scale. For concurrent validity we use *vaccination behavior* (vaccination status on an individual level) as a clear behavioral outcome [6]. As another consequence, using the 5C scale will not lead to a total score providing a sample’s absolute state of hesitancy. Rather, it will allow valid assessment of determinants predicting vaccination, allowing monitoring and evidence-informed intervention design.

### Existing measures and aims of the 5C scale

As a result of enduring efforts to define and measure vaccine hesitancy or confidence, there is a growing number of measures (right side of Table 1), such as the Parent Attitudes about Childhood Vaccines survey PACV [17], the Vaccine Confidence Scale VCS [18,29], a set of four items forming a Global Vaccine Confidence Index GVCI [4], the only recently published Vaccine Hesitancy Scale VHS [30,31], the Vaccine Acceptance Scale VAS [20] and the Vaccine Confidence Index VCI [21].

As can be seen in Table 1, most existing measures focus primarily on the confidence-related aspects of hesitancy and only occasionally go beyond this major factor (for a detailed description of the scales, see methods sections of Studies 2 and 3). However, a recent systematic review on influenza vaccine hesitancy has shown that the other constructs proposed in the hesitancy models are also important predictors of vaccination intention and behavior [11]. A tool that can assist in designing campaigns and interventions should therefore also assess complacency, constraints (former convenience), calculation, and collective responsibility. In addition, the existing scales are quite lengthy and use between 8 and 20 items to assess different facets of confidence (Table 1)–with the exception of the GVCI, which has only 4 items. A short measure with, at the same time, a broader scope will thus be useful both for research and practice. Finally, as previous studies showed that confidence is related to vaccine attitudes [31], a new measure should also relate each of the remaining concepts to psychological constructs [7] to demonstrate their validity and theoretical foundation.

Therefore, the 5C scale will provide a long and a short version to measure the 5C psychological antecedents of vaccination: confidence, complacency, constraints, calculation, and collective responsibility. In the validation process, we will relate the 5Cs to psychological constructs to understand the psychological underpinnings of vaccine uptake. The new scale will further need to demonstrate its added value in comparison with existing scales.

### The psychological antecedents of vaccination in the 5C scale

This section defines all five psychological antecedents of vaccination represented in the 5C scale and derives relations to validation constructs (for a summary, see Table 2). Please note that the fifth antecedent was added only after Study 1.

**Table 2:** Relations between 5C sub-scales and validation constructs.

	Confidence	Constraints	Complacency	Calculation	Collective responsibility
Vaccination behavior	(+)	(-)	(-)	(-)	(+)
Intention to vaccinate	(+)	(-)	(-)	(-)	(+)
**Study 1**	attitude (+)	perceived behavioral control (-)	risk attitude (+)	risk attitude (-)	
	knowledge (+)	self-efficacy (-)	considering future consequences (-)	numeracy (+)†	
	beliefs about medicine: benefits (+)	empowerment (-)	perceived risk of disease (-)	perceived risk of disease (-)	
	beliefs about medicine: harms (-)		normative beliefs (-)	perceived risk of vaccination (+)	
	conspiracy mentality (-)				
**Study 2**	attitude (+)	self-control (-)	perceived threat due to infectious diseases (-)	preference for deliberation (+)	communal orientation (+)
	knowledge (+)	perceived time pressure (+)	perceived personal health status (+)†	superstitious beliefs (-)†	collectivism (+)
	trust in health care systems (provider, payer, institution) (+)	perceived access to health care (-)	invulnerability (+)		individualism (-)†
	conspiracy mentality (-)†				empathy (+)

*Note*. (+) hypothesized positive relation; (-) hypothesized negative relation.

^†^ Correlation did not occur as expected for either the long or short version or both (see S5 Table and Table 3).

*Confidence* “is defined as trust in (i) the effectiveness and safety of vaccines, (ii) the system that delivers them, including the reliability and competence of the health services and health professionals, and (iii) the motivations of policy-makers who decide on the need of vaccines” [5] (p. 2). Individuals who lack confidence have negative attitudes towards vaccination (in contrast to the complacency and convenience types), which guide behavior. Misinformation, belief in conspiracies, and increased perceptions of vaccine-related risks contribute to the negative attitude. We therefore expect a positive correlation between confidence and attitudes toward vaccination [32], correct knowledge about vaccination [33], trust in the health care system [34], beliefs about benefits of medicines, and a negative correlation with beliefs about harms of medicines [35] and conspiracy mentality [36].

*Complacency* “exists where perceived risks of vaccine-preventable diseases are low and vaccination is not deemed a necessary preventive action” [5] (p. 2). Complacent individuals do not feel threatened by infectious diseases and thus have no impetus to change their prevention behavior [37]. As there is low involvement, general knowledge, awareness, and the level of active information searching are also low [38]. The preventive behavior is also not seen as the descriptive or injunctive norm in the society, therefore, we expect no relation to subjective norms [39]. Complacency should, however, be negatively related to perceived risks of diseases [40]. As prevention is a future-oriented behavior, we also expect a negative correlation with the consideration of future consequences [41]. As consequences in the future are not relevant, individuals high in complacency should also have a positive general risk attitude, indicating a preference for risk-seeking behaviors [42]. This could also be related to feelings of invulnerability [43] and a positive subjective personal health status.

As we share the criticism of the term convenience, we offer the term constraints instead. *Constraints* are an issue when “physical availability, affordability and willingness-to-pay, geographical accessibility, ability to understand (language and health literacy) and appeal of immunization service affect uptake” [5] (p. 3). Thus, structural and psychological barriers (access, a lack of self-control) are ‘gate-keepers’, impeding the implementation of vaccination intentions into behavior. Travel time or inconvenient procedures may also act as barriers. Perceiving constraints should therefore be related to a lack of perceived behavioral control [32] self-efficacy [44] and empowerment [45]. We expect positive correlations with perceived time pressure and daily hassles [46], and a negative correlation with perceived access to health care [47].

*Calculation* refers to individuals’ engagement in extensive information searching. We assume that individuals high in calculation evaluate risks of infections and vaccination to derive a good decision. Calculation should therefore be related to perceived vaccination and disease risks [40]. Engaging in cost-benefit calculations could be a sign of being risk-averse, thus, the correlation with risk-attitude should be negative [42]. Depending on the information sources that are used, high calculation can lead to non-vaccination due to the high availability of anti-vaccination sources, for instance, in the internet [48]. In general, we expect that the more information a person looks for, the more vaccine-critical sources will be obtained [49], also supported by a false-balance effect in the media (e.g., by providing an equal number of pro- and contra-vaccination experts even though in total there are many more pro-vaccination than contra-vaccination experts [50]). Thus, we predict a positive correlation with perceived vaccination risks. Individuals high in calculation should be rather risk-averse, i.e., their conscious and controlled processing leads us to assume that avoiding risks may be an important motivator [42]. This should also be associated with a more deliberative cognitive style of decision making [51] and less irrational thinking (superstitious beliefs; [52]). We will further explore the relation with numeracy [53].

We define *collective responsibility* as the willingness to protect others by one’s own vaccination by means of herd immunity [27]. The flip-side is the willingness to free-ride when enough others are vaccinated [27,54,55]. Collective responsibility should correlate positively with collectivism [55,56], communal orientation [57], and empathy [58]. It should correlate negatively with individualism [56]. Thus, people high in collective responsibility are willing to vaccinate in another person’s interest. Having low values can indicate that a person does not know about herd immunity or does not care or does not want to vaccinate for the benefit of others.

As is clear from the definitions there is no umbrella concept that embraces all antecedents. Thus, it does not seem theoretically justified to calculate a total score across all antecedents. We see the value of the 5C scale in briefly assessing five antecedents to explain vaccination behavior and to design and evaluate interventions to increase it.

Each antecedent represents individual preferences or psychological, mental representations of the environment the respondent lives in. Thus, political realities or inequality that erode trust in the health system could be a driver of low confidence for a person in one country. Accessing misinformation on the internet and sharing a social environment full of vaccine-critical parents could be a reason for low confidence for another person in another country. Likewise, perceived constraints could be a function of a lack of access, inappropriate service delivery or, for minority groups, a reluctance to get registered. Thus, it is important to note that the 5C antecedents provide insights in the individual, psychological antecedents and are not suitable to identify systems-related factors–beyond the effect they have on mental representations (e.g., of limited access).

### Overview

For the construction of the scale, we applied a factor-analytical approach and optimized internal consistency to reach relatively homogeneous dimensions [59]. Construct validity is considered at all stages of the developmental process and guides the process of item selection. The first study used two German convenience samples to select the items and develop the initial scale. Additionally, we assessed the sub-scales’ convergent, discriminant, and concurrent validity (i.e., correlations with similar and dissimilar psychological constructs and correlations with vaccination behavior). In the second study, a German sample representative regarding age, gender and parenthood answered the items with respect to vaccination in general and with respect to specific vaccinations. The specific vaccinations varied according to age and parental status of the participants (e.g., influenza vaccine for the elderly, measles (MMR) vaccine for parents of younger children, and human papilloma virus (HPV) vaccine for parents of older children). We compared the concurrent validity of general vs. specific assessments, i.e., the correlations with vaccination intentions. Additionally, we selected items for a short version of the scale that are as valid as the long version. Existing measures of vaccine hesitancy served as a benchmark to explore the added value of the new scale. Study 3 used a US convenience-sample of parents to compare the new scale to six existing scales and provides a validation of the English version of the scale. We used acceptance of the same vaccines to compare the scales’ concurrent validity (flu, HPV, MMR). S1 Table provides an overview of all studies. As an outcome, a long (15-item) and short (5-item) scale were developed as reliable and valid indicators of confidence, complacency, constraints, calculation, and collective responsibility.

## Study 1: Scale construction

Study 1 involved two samples to collect “responses to the initial set of items, generate and implement an item selection strategy, and construct provisional scales” [59]. Both samples received all items for the scale construction; the validation construct assessed in each sample varied between the samples (S2 Table).

### Method

#### Participants

This phase aimed to establish the content validity of the scale. We therefore recruited two initial convenience samples, i.e., an online convenience sample (*n* = 1,033, *M*_age_ = 32.92, *SD* = 9.37, 71% female; 495 were parents of at least one child; 86.92% completed the study) recruited via social media, and a student convenience sample (*n* = 412, *M*_age_ = 22.21, *SD* = 3.55, 83% female). Students were recruited via ORSEE, an online recruiting system [60]. As compensation, the participants in the convenience sample took part in a raffle for three 25 € gift vouchers; students took part in a lottery for 10 € coffee vouchers for the local campus coffee shop. There were no specified inclusion criteria. Data collection took place in Fall 2015. Sample size was determined by previous recommendations for sufficient power for scale construction [61] and for detecting small correlations (*r* = .15) with at least 85% power in the second subsample [62].

#### Item development

Based upon the theoretical definitions above, we developed a large item pool aiming for good content validity with “high relevance to the construct […] and representative of all potentially important aspects of the target construct” [59]. Additionally, existing measures were screened for suitable items. A final set of 35 items underwent factor and item analysis.

#### Vaccination behavior and intention

Vaccination behavior was measured as the sum of previously received vaccines (pertussis, tetanus, polio, diphtheria, measles, “don’t know” counted as missing values; score ranges between 0 and 5). The intention to vaccinate was measured by one item (“Imagine your next vaccine is due at your next GP routine visit. How would you decide?”; 1 = definitely not vaccinate, 7 = definitely vaccinate). These two constructs serve as major indicators of concurrent construct validity. It was expected that both constructs will be positively influenced by confidence, and negatively by complacency, constraints, and calculation.

#### Validation constructs and expected relations

The constructs were selected to assess whether the sub-scales are related to the intended psychological constructs [22], thereby assisting the process of item selection. Table 2 provides all hypotheses. We used validated and published measures where possible. S2 Table provides all constructs’ definitions, measurement, and respective references. The attitude toward vaccination, perceived behavioral control, and subjective norms were assessed following the Theory of Planned Behavior [39,63]. Correct knowledge about vaccination was assessed by a validated knowledge scale [33]. We further used validated scales to assess beliefs about benefits and harms of medicines [35], conspiracy mentality [36], consideration of future consequences [41], the general risk attitude [42], empowerment [45], self-efficacy [44], and numeracy [64]. Perceived risks of diseases and of vaccination were assessed by a single-item measure each (“How risky do you judge…”, scale 1–100) [65]. We also explored the correlations with social desirability [66] and the Big Five personality factors [67].

#### Procedure

The original online questionnaire is available at the online repository of the Open Science Framework (OSF; https://osf.io/agqem/). After providing informed consent and demographic information, participants filled in the items for the construction of the new scale, followed by the validation constructs, vaccination behavior, and vaccination intention. Within each scale the items occurred in randomized order.

#### Statistical analysis and item analysis

The procedure for data analysis follows the suggestions by [68]. We used common factor analysis with a maximum of 100 iterations for convergence to produce a homogenous set of well-interpretable factors. We used a pre-selection of four factors to select the items and then used Varimax rotation which allows for correlation of the factors as, theoretically, we did not assume orthogonality. We selected items with factor loadings of .5 and higher on the primary factor and minimal cross-loadings on any of the other factors (*a* < .35) to reduce overlap between the sub-scales. Internal consistency (reliability) was assessed by Cronbach’s α, which allowed further item selection by excluding items that substantially reduced α. To balance measurement precision and proper scale breadth, we allowed Cronbach’s α to be .70 or higher [59].

### Results

For data analysis, we recoded the items where necessary. Data and SPSS syntax are available for all studies via the OSF (https://osf.io/agqem/).

#### Exploratory factor analyses across both samples

The first factor analysis (*N* = 1,445; KMO = .93) using all items revealed seven factors with Eigenvalues > 1 (explaining 57.14% of the total variance). We extracted four factors and selected for each factor the three or four items with the highest factor loadings after Varimax rotation with no or low cross-loading on any of the other factors (S3 Table). With the selected 15 items, we repeated the factor analysis to explore the structure and the factor loadings. When inspecting the match between the selected items and the breadth of the theoretical concepts, we replaced one item in the confidence scale to also cover the aspect of trust in authorities rather than specific myths (elimination of: “It is better to strengthen one’s immune system through illness rather than by vaccination.” Replaced by: “Regarding vaccines, I am confident that public authorities decide in the best interest of the community.”). We further replaced “I understand how vaccines work” with “I decide based upon my feelings whether I should get vaccinated” in the complacency factor. Note that complacency was totally reworked in Study 2.

In a final factor analysis (S3 Table, right) the four factors explained 65% of the total variance. After Varimax rotation, the assumed pattern occurred with a Scree-Plot suggesting four factors, and with four items loading on confidence (e.g., “I am confident that routine vaccines are safe.”, Cronbach’s α = .88), four items loading on constraints (“Everyday stress prevents me from getting vaccinated.”, α = .77), four items loading on complacency (e.g., “I think there are as many reasons in favor of vaccination as there are against it.”, α = .67), and three items loading on calculation (e.g., “When I think about getting vaccinated, I carefully weigh the risks and benefits.”, α = .75). S4 Table provides all German items.

**Fig 1:**
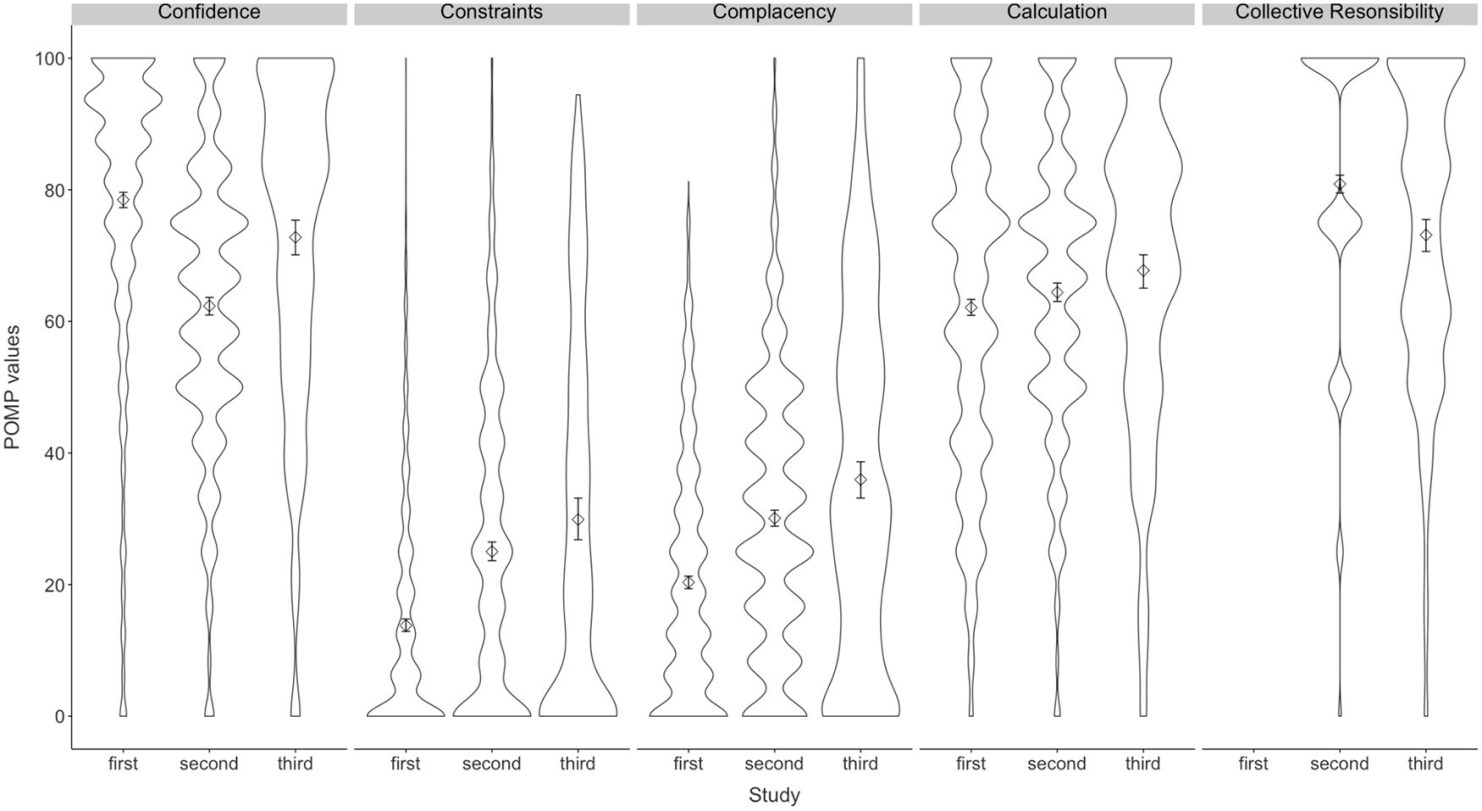
Violin plots of mean scores and distributions of the 5C antecedents of vaccination in Studies 1–3. The figure shows the means (diamonds) and 95% CIs (whiskers) and the frequency distribution of the 5C antecedents of vaccination across the three studies. Note that the items of the complacency and collective responsibility sub-scales are not identical across the studies. The exact wording of some items changed from Study 1 to Study 2 to increase item difficulty. The figure suggests that over the course of the development of the scale, the mean scores of the final scale (Study 3) are distributed more evenly across the possible spectrum, i.e., the items were not too “easy” or too “difficult” (e.g., as for constraints in Study 1, where the great majority of participants reported no constraints). Study 1: *N* = 1,445, Study 2: *N* = 1,003, Study 3: *N* = 350. The Y-axis shows POMP values: percent of maximum possible score [((observed score–minimum score)/(maximum score–minimum score)) x 100]. An increase of 1 unit on a POMP scale corresponds to an increase of 1% on the original scale. For example, an increase of 20 on the POMP scale corresponds to an increase of 1 original point of a 5-point scale. Collective responsibility was not measured in Study 1.

Fig 1 shows the mean values (diamonds) and distributions of the 5Cs across all three studies. The Y-axis shows POMP values: percent of maximum possible score [((observed score–minimum score)/(maximum score–minimum score)) x 100]. An increase of 1 on a POMP scale corresponds to an increase of 1% on the original scale. For example, an increase of 20 on the POMP scale corresponds to an increase of 1 original point of a 5-point scale. We chose POMP values as Study 3 uses a 7-point scale. Higher values indicate higher scores on the respective scale. Note that across studies the items per sub-scale and the samples differ, leading to the different distributions.

#### Concurrent validity

All constructs were significantly correlated with vaccination behavior and the future intention to get vaccinated (between *r* = -.14 and *r* = .84, all *p*s < .01) in the theoretically expected directions (positive for confidence, negative for constraints, complacency, and calculation).

#### Construct validity

For construct validity, we correlated the sub-scales with the validation constructs (Table 2; correlations S5 Table). Individuals with high confidence had a positive attitude (*r* = .82, all reported correlations were significant at *p* < .01), had more knowledge about vaccination (*r* = .81), believed that medicines have more benefits (*r* = .33) and fewer harms (*r* = -.35), and believed less in conspiracy theories (*r* = -.23). Beyond these predicted relations, we found that individuals high in confidence perceived higher risks of vaccine-preventable diseases (*r* = .56) and lower risks of vaccine adverse events (*r* = -.86), they considered vaccination as the social norm (*r* = .50), and based their decision less on experiences (*r* = -.15) and the opinion of others (*r* = -.28). Individuals perceiving constraints that keep them away from vaccinating reported lower behavioral control (*r* = -.21) and general self-efficacy (*r* = -.13). Correlations with the empowerment scales were rather weak (*r*s of .15 and 0).

Inspection of the complacency items revealed that the content of the resulting scale did not match the definitions of complacency. It contained items that mainly referred to the decision process (e.g., “I decide based upon my feelings.”; “I have never really thought about vaccines.”) instead of focusing on the low awareness of the disease risk and the importance of vaccination. Therefore, we decided to reconstruct this dimension in Study 2 and do not discuss the results of the correlation analysis here.

Individuals who have a high motivation for cost-benefit analysis had a low risk attitude (*r* = -.19) and were therefore rather risk averse. Their perception of disease risks (*r* = -.20) as well as of vaccination risks (*r* = .34) was significantly correlated with calculation. The correlation with knowledge was negative (*r* = -.28), i.e., extended information searching led to less valid knowledge. A potential cause for this could be that individuals high in calculation also based their decisions on the experiences of others, as shown by the positive correlation with empowerment (*r* = .22). Additionally, calculating individuals are rather conscientious (*r* = .21), which may explain the motivation for information searching in the first place.

All sub-scales were not significantly related to general measures of personality (with the noted exception of conscientiousness), numeracy, and social desirability (all *r*s < .15).

### Discussion of Study 1 and aims of Study 2

In Study 1, results of factor analyses allowed the selection of items that constitute four factors predicting vaccination behavior. For complacency, however, new items needed to be generated for Study 2, as the factor did not capture complacency as defined above and the internal consistency was sub-optimal. The calculation items were too similar, so we strived to broaden the concept by replacing one item.

As Fig 1 reveals, the item difficulty for the confidence and constraints sub-scales seems rather low (allowing many people to reach extreme scores). In order to avoid ceiling or floor effects, we reformulated items to make them more difficult (e.g., “I am confident that vaccines are safe.” changed to “I am *completely* confident that vaccines are safe.”). Additionally, we strived for an equal number of items per sub-scale. We will therefore test in Study 2 whether the long version can include 3 items per sub-scale while maintaining sufficient reliability.

The scale in its version after Study 1 does not include the awareness of the social benefit. The 5A taxonomy stresses its importance [14] and recent research has shown that people do care about others when making vaccination decisions [69–71]. Communicating the social benefit of vaccinations also leads to a higher willingness to vaccinate [55]. However, knowing about herd immunity may also instigate free-riding, as a well-vaccinated society may be sufficient to also protect unvaccinated individuals [72]. In order to capture the tendency to either vaccinate with pro-social intentions or to selfishly opt out, Study 2 added items to assess *collective responsibility*.

## Study 2: Refinement, validation and comparison to existing measures

Study 2 aimed to refine the content validity and reliability of the 5C scale as outlined above. This also involved further validation with psychological constructs. Additionally, we strived to compare the new scale to existing measures and to develop a shorter version of the scale.

The samples used in Study 1 were convenience samples of the general population and students. As vaccination is relevant in all age groups and may vary in importance depending on target or risk group (parents, elderly, travelers), we drew a sample representative of age, gender and parenthood for the German population. Moreover, we provided the 5C scale repeatedly: The first scale was directed at vaccination in general (as in Study 1). The second scale asked directly for specific vaccines (e.g., parents of younger vs. older children were asked with respect to measles vs. HPV vaccine, the elderly over 60 years were asked with respect to influenza, and travelers with respect to hepatitis A vaccination). This allows for comparison of general vs. specific measurement in order to assess whether it is sufficient to use a general scale or whether the antecedents vary in their values and relations to vaccination in a vaccine-specific way.

As we assume that the 5Cs (e.g., confidence) are related to psychological concepts (e.g., attitude, knowledge), in Study 2 we assessed further constructs to assess content validity. As outlined in the introduction, for confidence, we expected positive correlations with attitudes toward vaccination, knowledge about vaccination, and trust in health care providers. We expect negative relations between complacency and perceived risks of diseases and positive relations with perceived goodness of health status and invulnerability. Perceiving constraints should correlate positively with a lack of self-control and perceived everyday stress. Calculation should be positively correlated with preference for deliberation and conscientiousness. Lastly, the new factor collective responsibility should correlate positively with communal orientation, collectivism-individualism, and empathy. In Study 2, we also compared the 5C scale to existing measures (PACV, VCS) to assess its added value in predicting vaccination behavior.

### Methods

#### Participants

The recruited sample (*N* = 1,003) had a mean age of *M*_*age*_ = 47.98 (*SD* = 15.62), 15% were parents of a child between 11 months and 6 years of age, 16% had girls between 9 and 17 years of age, 31% of the participants were over 60 years of age, 29% had traveled in risk areas. Education was recoded according to the International Standard Classification of Education, yielding 17,5% in level 2 (lower secondary education), 58% in level 3 (upper secondary education), 23% in level 5 (short-cycle tertiary education) and 1.5% in level 6 (Bachelor’s degree or equivalent tertiary education level). The sample was representative regarding age, gender and parenthood of the German population (quotas: 25.22% parents; 48.55% males; 18.07% males aged 18–29; 15.2% males aged 30–39; 21.36% males aged 40–49; 17.25% males aged 50–59; 28.13 males with age > 59; 16.67% females aged 18–29; 13.95% females aged 30–39; 19.38% females aged 40–49; 16.28% females aged 50–59; 33.72 females with age > 59. These quotas are representative for the German population. Sampling took place until the quotas were reached).

Data collection took place in Spring 2017. All participants were recruited and took part in the study online via a recruitment agency (using an ISO-certified panel–ISO 9001/26362), from which they received compensation (bonus points to exchange into money).

#### Item development

In order to create a new complacency sub-scale, we developed five item candidates (e.g., “Vaccination is unnecessary because vaccine-preventable diseases are not common anymore.”). We further defined collective responsibility as the willingness to protect others by one’s own vaccination. The flip-side is the willingness to free-ride when enough others are vaccinated [27,55]. We generated three items to capture this aspect. In order to reduce redundancy in the calculation sub-scale, we added two additional item candidates in order to exchange one of the items.

#### Existing measures

This study included the two most established measures of vaccine hesitancy, the Parent Attitudes About Childhood Vaccines survey (PACV; [17] and the Vaccine Confidence Scale (VCS; [29]).

**PACV**. The PACV [17] assesses vaccination attitudes (e.g., “Overall, how hesitant about childhood shots would you consider yourself to be?”), beliefs about vaccine safety and effectiveness (e.g., “How concerned are you that your child might have a serious side effect from a shot?”), and behavior (e.g., “Have you ever decided not to have your child get a shot for reasons other than illness or allergy?”). All three concepts are part of the confidence construct (Table 1). The scale uses six different answer formats (e.g., agree/disagree/not sure, yes/no/don’t know, 1–10 rating scales). All answers are recoded in the three categories hesitant, non-hesitant and not sure/don’t know. The sub-scales have good reliability (Cronbach’s α between .74 and .84, [17]). All 15 items can be summarized to a total score which predicts under-immunization of children [17,73,74]. The scale has been used several times, also to detect the impact of interventions [75–77]. Additionally, there is a modified version to match influenza vaccination [73]. A short version has been used recently [78]; translations are available for Italian [79] and Malay [80].

**VCS**. The VCS [29] is based on the Health Belief Model and assesses vaccine confidence with three sub-scales: benefits (e.g., “Vaccines are safe.”), harms (e.g., “Teenagers receive too many vaccines.”), and trust (e.g., “I have a good relationship with my teenager’s health care provider.”). Again, all three sub-scales assess parts of the confidence construct (Table 1). The items are rated on an 11-point scale, ranging from 0 (strongly disagree) to 10 (strongly agree); the internal consistency of the sub-scales is low to acceptable (Cronbach’s α between .49 and .78). The authors criticized existing measures to be only directed at specific vaccinations [81] or populations [17] and therefore developed a measure “capable of characterizing adolescent vaccination beliefs more holistically” to “perform reliably across diverse populations” [29]. In the present study we re-formulated the items to eliminate the explicit relation to teenagers (e.g., replacing ‘teenagers receive too many vaccines’ with ‘people receive too many vaccines’). The benefit sub-scale (4 items) can be used as a short version of the scale. The confidence index, calculated as the mean of all 8 items, predicts vaccine uptake and refusal in parents of small children [82] and adolescents [18].

#### Vaccination behavior and intention

General vaccination behavior was again assessed as the sum of previously received recommended vaccines (yes/no/don’t know; regarding pertussis, tetanus, polio, diphtheria, influenza, measles, hepatitis A, hepatitis B, varicella, pneumococcus, meningococcus C; don’t know was counted as missing value, sum ranging between 0 and 11). Parental vaccination behavior was assessed as having vs. not having vaccinated the child against measles or HPV, target group-specific acceptance is represented by having received influenza (when over 60 years of age) or having received hepatitis A (when traveler).

#### Additional validation constructs and expected relations

As in Study 1, Table 2 provides hypotheses; S2 Table gives details on all constructs’ definitions, measurement, and respective references. All constructs were assessed by validated and published scales. The attitude towards vaccination was assessed following the Theory of Planned Behavior [39,63]. Correct knowledge about vaccination was gathered with a validated knowledge scale [33]. We further used validated scales to measure conspiracy mentality [36] and empowerment [45]. Perceived risks of diseases and of vaccination were assessed by a single-item measure each (“How risky do you judge…”, scale 1–100 [65]). We also explored the correlations with a short Big Five personality factors scale [67]. Additionally, trust in health care systems was collected [34]. Perceived health status was surveyed with the General Health Perceptions scale [83]. Likewise, we assessed individuals’ general vulnerability [43], the preference for deliberation [51], superstitious beliefs [52], self-control [84], time pressure [46], communal orientation [57], collectivism [56], and empathy [58].

#### Procedure

The original questionnaire is available at the OSF (https://osf.io/agqem/). First, respondents were asked to indicate their demographics (gender, age, highest level of education), whether they have children between 11 months and 6 years of age, a daughter between 9 and 17 years of age, whether they were chronically ill, worked in the healthcare sector, live in a more rural or urban area, and whether they have traveled to Southeast Europe, Asia, Africa, Middle or South America (these regions are considered high-risk regions for diseases such as hepatitis A) in the last five years. Parts of these questions were used as quotas for the representative sampling and to ensure a target group-specific survey. Then, we assessed vaccination behavior. Parents additionally indicated the vaccination status of their children. Afterwards, the 5C items and vaccination attitude were measured. The battery was provided more than once and adapted to specific infectious diseases when the participants belonged to the following subgroups, referring to the vaccination schedule in Germany [85]: we provided parents of young children between 11 months and 6 years of age with extra questions regarding the measles vaccine, parents of girls between 9 and 17 years of age with items regarding the human papilloma virus (HPV) vaccine, elderly above 60 years of age, health care personnel, or chronically ill individuals with items regarding influenza vaccine, and participants who had traveled to regions where travel vaccinations are recommended received items on the hepatitis A vaccine. Then, the validation constructs were assessed: knowledge, PACV, VCS, empowerment, trust in health care systems, risk perceptions of diseases (measles, HPV, influenza contingent upon target group), perceived health status, perceived access to health care, invulnerability, preference for deliberation, conspiracy mentality, superstitious beliefs, self-control, time pressure, communal orientation, collectivism/individualism, empathy, and the Big Five personality factors short scale.

#### Statistical analysis

Cronbach’s α was used as an indicator of reliability; correlation analysis was used for assessing the concurrent and construct validity as well as for the comparison to existing measures. Linear regression analysis (stepwise) was used to predict vaccination behavior and binary logistic regression to predict single vaccinations.

### Results

For data analysis, we recoded the items where necessary. Data and SPSS syntax (SPSS 25) are available at the OSF (https://osf.io/agqem/).

#### Item analysis

Table 3 displays the pre-final 5C scale in a long and short version (note that the final version is presented below as a result of Study 3). Bold items constitute the final short version. Three items assessed confidence with a reliability of α = .80. Fig 1 reveals that the overall item difficulty was higher, as indicated by a lower mean score. This was intended by the change to more extreme wording. Reliability of the three items assessing constraints was also very good (α = .81). The five complacency items had an initial Cronbach’s α of .69; in a stepwise elimination process we eliminated the two items that decreased Cronbach’s α, leading to a 3-item sub-scale with acceptable α = .75. After the same procedure, the five calculation items reduced to three items with α = .80. Collective responsibility did not turn out to be a reliable sub-scale, Cronbach’s α was .47 for the three items. Thus, we decided to include collective responsibility as a single-item measure at this stage. We chose the item that showed best performance in the validation for the short measure (below).

**Table 3:** Items of a pre-final 5C scale and Pearson correlations of the long and single-item short version of the 5C scale with validation constructs (Study 2).

5C sub-scale and items	Validation construct	Long version	Single-item version
**Confidence (α = 0.80)**			
**I am completely confident that vaccines are safe**.	attitude	0.78	0.72
Vaccinations are effective.	knowledge	0.47	0.45
Regarding vaccines, I am confident that public authorities decide in the best interest of the community.	trust in provider	0.46	0.41
	trust in payer	0.31	0.27
	trust in institutions	0.32	0.29
	conspiracy mentality	-.07*	-.05 ns
**Constraints (α = 0.81)**			
**Everyday stress prevents me from getting vaccinated**.	self-control	-0.37	-0.31
For me, it is inconvenient to receive vaccinations.	time pressure	0.23	0.26
Visiting the doctor’s makes me feel uncomfortable; this keeps me from getting vaccinated.	access to health care	-0.17	-0.13
**Complacency (α = 0.75)**			
**Vaccination is unnecessary because vaccine-preventable diseases are not common anymore**.	perceived threat of VPD	-0.28	-0.21
My immune system is so strong, it also protects me against diseases.	personal health status	0.16	.01 ns
Vaccine-preventable diseases are not so severe that I should get vaccinated.	invulnerability	0.47	0.39
**Calculation (α = 0.80)**			
**When I think about getting vaccinated, I weigh benefits and risks to make the best decision possible**.	preference for deliberation	0.3	0.25
For each and every vaccination, I closely consider whether it is useful for me.	superstitious beliefs	.02 ns	.04 ns
It is important for me to fully understand the topic of vaccination, before I get vaccinated.			
**Collective responsibility (α = n.a.)**			
**When everyone is vaccinated, I don’t have to get vaccinated, too**.	communal orientation	n.a.	0.35
	collectivism		-.07*
	empathy		0.37
	individualism		.01 ns

Bold items represent the items from the short version of the 5C scale. All *p*s < .001, except *, which are significant at *p* < .05. ns not significant. VPD = vaccine-preventable diseases. Note that Table 5 presents the final version of the 5C scale.

#### Construct validity

All relevant psychological constructs were significantly related to confidence as hypothesized (attitude, knowledge, (weakly) conspiracy mentality and trust; Table 2). Constraints were significantly related to self-control, perceived time pressure, and perceived access to health care. Complacency was significantly correlated with perceived threat of measles, personal health status, and invulnerability. For calculation, only preference for deliberation was a significant correlation. Superstitious beliefs were not related to calculation. Finally, collective responsibility was positively related to communal orientation, collectivism, and empathy. Individualism was not significantly related to collective responsibility.

#### Validation of the short scale

The development of the short scale is based on another representative sample and will be described elsewhere. As a result of that process, for each sub-scale one representative item was selected. The short and long scales were correlated with the validation constructs assessed in this study to compare the patterns and to assess the validity of the short scale. Table 4 provides the results, which show that both the single-item short scale and the long scale correlate in the expected directions and to a similar extent as the validation constructs. The only exception is personal health status, which shows a low correlation with complacency in the 3-item version, but none in the 1-item version. In total, the correlations are generally somewhat weaker, but still significant. We therefore assume that the short version’s validity is similar to the long one.

**Table 4:** Results of six binary logistic regressions to compare the explanatory value of the general and specific 5C scale in predicting acceptance of MMR, HPV and flu vaccination (Study 2).

	MMR vaccination of children below 6 y/a (*n* = 144; 93.1% vaccinated)						HPV vaccination of daughter between 9 and 13 y/a (*n* = 154; 52.6% vaccinated)						Flu vaccination of participants over 60 y/a (*n* = 543; 37.4% vaccinated)					
	5C general			5C MMR-specific			5C general			5C HPV-specific			5C general			5C Flu-specific		
	*B*	*SE*	*OR*	*B*	*SE*	*OR*	*B*	*SE*	*OR*	*B*	*SE*	*OR*	*B*	*SE*	*OR*	*B*	*SE*	*OR*
Confidence	**0.930**	**0.423**	**2.536**	**1.225**	**0.454**	**3.404**	**0.781**	**0.235**	**2.183**	**0.951**	**0.230**	**2.589**	**0.921**	**0.159**	**2.513**	**1.609**	**0.184**	**4.999**
Constraints	0.649	0.541	1.913	0.400	0.575	1.492	-0.067	0.258	0.935	0.261	0.323	1.298	**-0.417**	**0.168**	**0.659**	**-0.674**	**0.199**	**0.509**
Complacency	-0.454	0.496	0.635	-0.164	0.495	0.849	-0.050	0.28	0.951	0.535	0.339	1.708	**-0.581**	**0.191**	**0.56**	**-0.786**	**0.196**	**0.456**
Calculation	-0.743	0.531	0.476	-0.583	0.472	0.558	0.309	0.238	1.363	0.062	0.228	1.064	**-0.309**	**0.127**	**0.734**	**-0.388**	**0.158**	**0.679**
Coll. Resp.	-0.071	0.391	0.932	-0.275	0.534	0.760	-0.452	0.256	0.636	0.38	0.336	1.463	-0.202	0.176	0.817	**-0.514**	**0.199**	**0.598**
Gender	0.775	0.814	2.171	0.788	0.856	2.199	-0.062	0.361	0.939	-0.188	0.378	0.829	-0.347	0.22	0.706	-0.293	0.260	0.746
Age	-0.026	0.042	0.975	-0.002	0.043	0.998	-0.031	0.02	0.969	-0.031	0.020	0.969	**0.034**	**0.008**	**1.035**	0.017	0.009	1.017
Education	0.241	0.417	1.272	0.337	0.405	1.400	-0.148	0.189	0.863	0.006	0.190	1.006	0.146	0.099	1.157	**0.251**	**0.118**	**1.285**
Constant	1.684	4.614	5.386	-0.697	4.629	0.498	0.212	2.405	1.236	-5.117	2.825	0.006	-1.884	1.446	0.152	-1.136	1.544	0.321
Nagelkerke’s *R*^2^	0.309			0.305			0.169			0.224			0.352			0.579		

The pattern of results remained stable when not controlling for age, gender and education. Bold coefficients are significant at *p* < 0.05; all other ns.

When predicting vaccination behavior, we used the long version as well as the short version of the 5C scale in a stepwise regression analysis. The construction of a new scale is based on the idea that existing scales mainly assess confidence and that additional constructs need to be measured, too. Therefore, in the first step we entered confidence, then the remaining four constructs to assess their added value; in a third step we controlled for age, gender and level of education. For both the long and short scale the pattern was equal: confidence, constraints, and complacency were significant predictors (long scale: βs = .22, -.14, -.11; all *p*s < .01; short scale: β = .27, *p* < .01; -.10, -.08; *p*s < .05, respectively), while calculation and collective responsibility were not significant predictors. *R*^2^ for the full models were also similar (corrected *R*^2^ = .15 for the long and .15 for the short scale). The change in explained variance by adding constraints, complacency, calculation and collective responsibility was significant in both cases and increased from 8% (long) and 9% (short) to 11%. These results show the usefulness of extending the prediction of vaccination beyond confidence.

#### Comparison to existing measures

As the existing scales measure confidence, we analyzed whether they correlate with the newly constructed 5C confidence sub-scale. Correlation analysis (S6 Table) shows that both PACV and VCS correlate significantly and highly with confidence (PACV: *r* = -.51; VCS: *r* = .77, *p*s < .001). Additionally, both scales similarly correlate with complacency (*r*s = .60, -.59, respectively, *p*s < .001), suggesting that PACV and VCS also assess the perception of the disease risk. All other sub-scales correlate with the PACV and VCS between -.15 and .40 (*p*s < .001), indicating sufficient difference between the 5C antecedents of vaccination and the existing scales.

#### Predicting vaccination behavior: General vs. specific measurement of the 5Cs

In four sub-populations (parents of a child aged < 6 years; parents of a daughter between the age of 9 and 13, travelers in risk regions and the elderly), we assessed the 5C scale twice: the general one discussed so far, and a scale in which each item related to a specific vaccination (e.g., “Everyday stress prevents me from getting vaccinated.” vs. “Everyday stress prevents me from getting vaccinated *against influenza*.”). We calculated all sub-scales and regressed specific vaccination behavior on the 5C sub-scales. Specific vaccination behavior is a binary variable, indicating whether a participant’s child below 6 years of age had received the MMR or the daughter between 9 and 13 years of age had received the HPV vaccine, or whether the elderly had received the flu vaccine. As all participants with travel experience in risk regions were vaccinated against hepatitis A, we did not calculate any regressions here. Table 3 displays the results. Overall, there was no general advantage for the specific scale. The amount of explained variance increased in two of the three cases (HPV, flu) when measured specifically but remained similar in the third case (MMR). The pattern of significant predictors was more or less independent from the general vs. specific wording: for MMR and HPV vaccine, confidence was the only predictor in both models; for influenza vaccine, all antecedents were significant predictors when measured in a specific way; in the general model, collective responsibility was not significant. Thus, while the pattern of relevant predictors seems to vary between the different vaccinations, the overall pattern of relevant predictors, however, can be captured by a general measure as well.

### Discussion and aims for Study 3

Study 2 demonstrates the reliability and validity of the proposed antecedents of vaccination, except for the sub-scale collective responsibility. The correlations with other constructs were in the expected directions. This yields progress in being more specific and theoretically precise when assessing psychological antecedents of vaccination [11,12,22].

Unfortunately, the collective responsibility sub-scale resulted in only one item, as the 3-item solution was not reliable. Thus, Study 3 will add two additional items to the collective responsibility sub-scale, so that each sub-scale has the same number of items. The short version turned out as valid as the long version, which allows the future use of one item per sub-scale (e.g., in field settings or for monitoring) without losing validity. When comparing the 5C scale with existing scales, the results showed that there is considerable overlap with the 5C confidence sub-scale and existing scales that assess confidence (PACV, VCS; see also Table 1) and sufficient distinctiveness from the existing scales when looking at the other sub-scales. Generally, there was no overarching advantage of using a vaccine-specific version. As a limitation, it is important to note that the uptake for MMR was very high in the sample (93.1%), so there was not much variance in the dependent variable. This may have reduced the predictive power of the scales.

## Study 3

In Study 3, the crowd-working platform Mechanical Turk (MTurk) was used for collecting data. We used a US sample of parents and assessed the 5C scale to also compare it with the measures that had been developed concurrently (Table 1), and to refine the final 5C scale. Further, using participants from the US allowed providing a validated English translation of the 5C scale. Most importantly, we extended the collective responsibility factor as it only had one item.

### Methods

#### Participants

Sample size was optimized for detecting small correlations (*r* = .2) with at least 95% power [62]. The parents were recruited and took part in the study online. 79.01% of the participants who started the study finished it, resulting in *N* = 350 parents. They received financial compensation via MTurk; US$2.50 for about 16 minutes completion time on average (*SD* = 12.8). Of the parents, 49% were female; *M*_age_ = 34.01, *SD* = 7.49; 92% were parents of a child equal to or above 2 years of age, 28% had children equal to or above 11 years of age. Education levels were recoded following the International Standard Classification of Education, yielding *n* = 3 participants at level 2 (lower secondary education), *n* = 101 on level 3 (upper secondary education), *n* = 242 on level 5 (short-cycle tertiary education), and *n* = 3 on level 6 (Bachelor’s degree or equivalent tertiary education level). In order to check the data quality in the MTurk sample we conducted two attention checks [86]. Twenty individuals failed on at least one of two attention checks. Excluding these from the analyses did not alter the results and thus we included all participants in the reported analyses. Data collection took place in Spring 2018.

#### 5C and further item development

As Study 2 revealed that for collective responsibility the available items were not suitable to build a reliable sub-scale, we constructed 11 new candidates, all verbalizing the idea that free-riding (reverse) or ensuring community/herd immunity is a good idea (e.g., “Ensuring community immunity is an important reason for me to get vaccinated.”). For the 5C scale, we decided to switch to a 7-point fully-labelled rating scale to increase the potential variance and allow for more fine-graded ratings (strongly/moderately/slightly disagree, neutral, slightly/moderately/strongly agree).

#### Existing measures

Six existing measures were included in this study. In addition to the two measures described in Study 2 (PACV, VCS), we included the scales below.

**GVCI**. The global vaccine confidence index [4] uses four items to assess whether vaccines are perceived as safe, important, effective, and compatible with religious beliefs (4-point scale, disagree to agree). The measure is based upon the ten-question Likert-type rating scale survey proposed by SAGE [30]. The authors see their scale as a global monitoring tool. It has been applied in multiple countries; the data are publicly available (www.vaccineconfidence.org). While safety and effectiveness are aspects of confidence, importance pertains to complacency and compatibility with religious beliefs is a new aspect that is not covered in the existing theoretical models (Table 1; see also General discussion).

**VHS**. The Vaccine Hesitancy Scale uses the same basic set of items as the GVCI, but it assesses the validity and reliability of the full 10 items as suggested in the original paper (5-point Likert-type rating scale ranging from strongly disagree to strongly agree). As the authors note, there has never been a psychometric validation since the items were proposed. Factor analyses revealed two factors: lack of confidence (e.g., “All childhood vaccines offered by the government program in my community are beneficial.” (reverse); α = .92) and risk due to vaccination (e.g., “New vaccines carry more risks than older vaccines.”; α = .64). The sub-scales were substantially correlated with the Vaccine Conspiracy Beliefs Scale [19] and the harms and benefits sub-scales of a hesitancy measure which is specifically related to HPV vaccination [87]. Concurrent validity was also supported by participants who had refused HPV vaccination showing higher hesitancy scores [31]. Thus, as indicated in Table 1, this scale is another measure that validly and reliably assesses the theoretical component of confidence.

**VAS**. The “survey instrument for measuring vaccine acceptance” (in the remainder referred to as the Vaccine Acceptance Scale; VAS) [20] criticizes existing measures as being theoretically inconsistent, using different numbers of items per sub-scale, using too many different and unbalanced item stems and scoring rules. In their new construction, they developed four items each for five reliable sub-scales: perceived safety of vaccines (e.g., “Vaccines contain mercury in dangerous amounts.” (reverse); α = .91), perceived effectiveness and necessity of vaccines (e.g., “Vaccines are effective at preventing diseases.”; α = .82), acceptance of the selection and scheduling of vaccines (e.g., “We give children the right number of vaccines.”; α = .89), positive values and affect toward vaccines (e.g., “I’m morally opposed to vaccinating my child.”; α = .91), and perceived legitimacy of authorities to require vaccinations (e.g., “The government should not force children to get vaccinated to attend school.”; α = .89). A total score (mean of all sub-scales; α = .96) and a short version (10 items) can also be calculated. According to the VAS validation data, acceptance is related to trust in biologists, conspiratorial thinking, and political ideology (higher acceptance in more liberal individuals). Thus, as indicated in Table 1, this scale is another measure that strongly relates to the theoretical concept of confidence.

**VCI**. The Vaccine Confidence Index VCI [21] aims to track changes in parents’ confidence over time as well as to assess confidence in provider settings. It is based on experts’ opinions and after item analysis it results in three constructs, i.e., trust (e.g., in the Food & Drug Administration (FDA), the federal government agency that licenses vaccines; four items, 7-point scale), importance (“It is important for everyone to get the recommended vaccines for their child(ren).”; one item, 5-point scale), confidence (e.g., “Vaccines recommended for young children are safe.”; three items, 6-point scale). As the sub-scales’ scoring varies between 5-, 6-, and 7-point scales, we calculated POMP values [88] before calculating a mean total score. The scale was obtained during an online search, to our understanding this is a scale in development. However, we decided that including this new measure would be useful for screening the market and to compare the 5C scale with existing and emerging approaches. As the title of the scale suggests, the VCI is a further candidate in assessing confidence with several sub-scales.

#### Vaccination behavior and intention

General vaccination behavior was again assessed as the sum of previously received recommended vaccines; yes/no/don’t know; don’t know answers were counted as missing values, sum ranging between 0–6 for adults (tetanus, diphtheria, pertussis, flu, varicella, MMR), and 0–11 for children (tetanus, diphtheria, pertussis, flu, varicella, MMR, Hepatitis A, B, haemophilus influenza type B, pneumococcal, polio, rota) as these were the vaccines recommended for adults and children, respectively [89]. Vaccination behavior for specific vaccinations was assessed as having vs. not having vaccinated the child against measles (children > 2 years of age) or HPV (children > 11 years of age), having received influenza was assessed for all participants as in the US there is a universal recommendation.

#### Additional validation constructs and expected relations

Communal orientation and social desirability were assessed as in Study 1 and 2 to validate the new 5C sub-scale collective responsibility and to check for its relation to socially desirable response tendencies.

#### Procedure

The original questionnaire is available at the OSF (https://osf.io/agqem/). After informed consent, the questionnaire first assessed age, gender, highest level of education, whether participants had children under 18 years of age, the age of their (up to five) oldest children if applicable, whether they were chronically ill, whether they were health care personnel, whether they lived in a more rural or urban area, and whether participants have traveled to Southeast Europe, Asia, Africa, Middle or South America in the last five years. After an attention check [86], we assessed vaccination behavior as a self-report of their own as well as their oldest child’s vaccination status. Then, the seven vaccination measures were assessed in random order and the sequence of the items within each measure was randomized, too. This was followed by a second attention check [86], communal orientation and social desirability, also in random order and with a random sequence of items within each measure. On the final page after the debrief, participants received a link to the CDC website on immunization for further information.

#### Statistical analysis

Cronbach’s α was used as an indicator of reliability; correlation analysis was used for assessing the construct validity as well as for the comparison to existing measures. Binary logistic regressions predict single vaccinations.

### Results

#### Item analysis and validation

In order to find items that complement the existing collective responsibility item, we chose items that matched the construct best and correlated with the validation construct communal orientation. Based on the reliability analyses we chose two additional items (“I get vaccinated because I can also protect people with a weaker immune system.”, “Vaccination is a collective action to prevent the spread of diseases.”) The sub-scale’s Cronbach’s α was .71; its correlation with communal orientation was *r* = -.17, *p* < .001; for downplaying negative qualities (social desirability) *r* = -.32, *p* < .001, and .09, ns, for emphasizing positive qualities. Table 5 presents the final resulting scale including all Cronbach’s αs. All sub-scales had sufficient reliability.

**Table 5:** The final English and German 5C scale measuring psychological antecedents of vaccination (Study 3).

English version	German version
Confidence α = .85	
**I am completely confident that vaccines are safe**.	**Ich habe vollstes Vertrauen in die Sicherheit von Impfungen**.
Vaccinations are effective.	Impfungen sind effektiv.
Regarding vaccines, I am confident that public authorities decide in the best interest of the community.	Was Impfen anbelangt, vertraue ich darauf, dass staatliche Behörden immer im besten Interesse für die Allgemeinheit entscheiden.
Complacency α = .76	
**Vaccination is unnecessary because vaccine-preventable diseases are not common anymore**.	**Impfungen sind überflüssig, da Krankheiten, gegen die man sich impfen lassen kann, kaum noch auftreten**.
My immune system is so strong, it also protects me against diseases.	Mein Immunsystem ist so stark, es schützt mich auch vor Erkrankungen.
Vaccine-preventable diseases are not so severe that I should get vaccinated.	Krankheiten, gegen die man sich impfen lassen kann, sind nicht so schlimm, dass ich mich gegen sie impfen lassen müsste.
Constraints α = .85	
**Everyday stress prevents me from getting vaccinated**.	**Alltagsstress hält mich davon ab, mich impfen zu lassen**.
For me, it is inconvenient to receive vaccinations.	Es ist für mich aufwändig, eine Impfung zu erhalten.
Visiting the doctor’s makes me feel uncomfortable; this keeps me from getting vaccinated.	Mein Unwohlsein bei Arztbesuchen hält mich vom Impfen ab.
Calculation α = .78	
**When I think about getting vaccinated, I weigh benefits and risks to make the best decision possible**.	**Wenn ich daran denke, mich impfen zu lassen, wäge ich Nutzen und Risiken ab, um die bestmögliche Entscheidung zu treffen**.
For each and every vaccination, I closely consider whether it is useful for me.	Ich überlege für jede Impfung sehr genau, ob sie sinnvoll für mich ist.
It is important for me to fully understand the topic of vaccination, before I get vaccinated.	Ein volles Verständnis über die Thematik der Impfung ist mir wichtig, bevor ich mich impfen lasse.
Collective responsibility α = .71	
**When everyone is vaccinated, I don’t have to get vaccinated, too. (R)**	**Wenn alle geimpft sind, brauche ich mich nicht auch noch impfen zu lassen. (R)**
I get vaccinated because I can also protect people with a weaker immune system.	Ich lasse mich impfen, weil ich auch Menschen mit einem schwachen Immunsystem schützen kann.
Vaccination is a collective action to prevent the spread of diseases.	Impfen ist eine gemeinschaftliche Maßnahme, um die Verbreitung von Krankheiten zu verhindern.

Instruction: “Please evaluate how much you disagree or agree with the following statements.” (1 = strongly disagree, 2 = moderately disagree, 3 = slightly disagree, 4 = neutral, 5 = slightly agree, 6 = moderately agree, 7 = strongly agree). Scoring: mean scores of each sub-scale. Item with (R) is reverse-coded. For the short scale use bold items. Cronbach’s α refers to the English version. The German translation of the collective responsibility scale has not been tested on a German sample yet.

#### Comparison with existing measures

S1 Fig provides the existing measure’s mean scores with 95% CIs. Inspection of the means shows that these are at significantly different levels; i.e., classifying samples according to the absolute values reached on different scales would lead to different interpretations with the VCS and GVCI testifying the highest confidence/acceptance and the VAS showing lowest levels of confidence/acceptance. Table 6 provides Cronbach’s αs and correlations between the 5C sub-scales and all hesitancy/acceptance measures. All existing scales correlate significantly and highly (> .6) with the 5C sub-scales confidence and collective responsibility. Additionally, PACV and VAS correlate highly with complacency. The lowest correlations of existing scales and the 5C sub-scales are with constraints and calculation, pointing out unique features of the 5C scale. The correlational pattern thus demonstrates good convergent and discriminant validity.

**Table 6:** Pearson zero-order correlations between the 5C sub-scales and all seven assessed hesitancy/acceptance measures (Study 3).

	Parental Attitudes about Childhood Vaccines	Vaccine Confidence Scale (benefit factor)	Global Vaccine Confidence Index	Vaccine Hesitancy Scale	Vaccine Acceptance	Vaccine Confidence Index
	Opel PACV	Gilkey VCS	Larson GVCI	Shapiro VHS	Sarath-chandra VAS	Frew VCI
*min*. *max*	[0,30]	[1,11]	[1,5]	[1,5]	[1,7]	[1,5;6;7]
5C Conf. α = .85	-.674**	.790**	.782**	.800**	-.764**	.828**
5C Constr. α = .85	.467**	-.308**	-.254**	-.440**	.547**	-.290**
5C Compl. α = .76	.619**	-.477**	-.414**	-.577**	.701**	-.429**
5C Calc. α = .78	.272**	-.093	-.084	-.172**	.237**	-.153**
5C Coll. Resp. α = .71	-.657**	.751**	.696**	.780**	-.765**	.692**
Total 5C α = .71	-.731**	.609**	.546**	.711**	-.806**	.600**
PACV α = .89		-.721**	-.689**	-.826**	.879**	-.732**
VCS α = .90			.835**	.875**	-.803**	.860**
GVCI α = .87				.823**	-.765**	.831**
VHS α = .90					-.894**	.874**
VAS α = .95						-.804**
VCI α = .95						-

The VHS scale is actually meant to be a 2-factor scale that is not combined. Cronbach’s alpha for the sub-scales were .76 for risks and .94 for lack of confidence. Recoding the two risk-items led to excellent Cronbach’s α. Therefore, we use the combined score here.

* *p* < .05;

** *p* < .01.

In order to compare the scales’ performance in predicting vaccination behavior, we conducted binary logistic regression analyses to predict whether adults had received flu vaccination, and the oldest child had received the MMR vaccine (for children ≥ 2 years of age, *n* = 320) and/or the HPV vaccine (for children ≥ 11 years of age, *n* = 97). As predictors, we used the respective sub-scales to predict specific vaccination behavior, controlled for age, gender and education.

The analysis was performed for each measure and each vaccine (Table 7), allowing comparison of the proportion of variance explained by the sub-scales and, for the existing measures, also for the total score (Nagelkerke’s *R*^2^). Analyses reveal that for all scales, between the vaccines, the sub-scales that predict acceptance vary. That is, predictors which are relevant for flu are not necessarily relevant for uptake of the MMR vaccine. Additionally, especially for the MMR vaccine, the significant predictors go beyond confidence, as other sub-scales are significantly related to acceptance as well (constraints, calculation). Moreover, the total amount of variance explained varies depending on the vaccination and the scale used to predict uptake. For flu vaccination, the maximum percentage of explained variance was 22% (VCI). For MMR and HPV vaccination, the highest proportion of explained variance was 42% (VAS) and 40% percent (VCI), respectively. In all cases the 5C scale consistently explained nearly as much variance (21% for flu, 40% for MMR and 35% for HPV). Thus, for all assessed vaccines the 5C scale constantly performed as good as the best performing scale, which varied for the different vaccines. Thus, we recommend using the 5C scale as a 5- or 15-item measure to assess the five psychological antecedents of vaccination to separately predict vaccination behavior.

**Table 7:** Regressions predicting vaccine acceptance (own flu vaccination, child’s MMR and HPV vaccination) by the sub-scales and total scores of all assessed measures (Study 3).

	Own flu vaccination *n* = 316 in the regression 48.0% vaccinated (168 out of 317)				Child’s MMR vaccination (child ≥ 2 yrs) *n* = 301 in the regression 83.0% vaccinated (268 out of 302)				Child’s HPV vaccination (child ≥ 11 yrs) *n* = 97, 53.6% vaccinated (52 out of 88)			
	*B*	*SE*	*OR*	*R*^*2*^	*B*	*SE*	*OR*	*R*^*2*^	*B*	*SE*	*OR*	*R*^*2*^
**5C**												
**sub-scales**				0.21				0.40				0.35
Confidence	**0.530**	**0.121**	1.698*		0.281	0.212	1.324		**0.759**	**0.256**	**2.137**	
Constraints	-0.085	0.109	0.919		**-0.361**	**0.169**	**0.697**		0.208	0.267	1.231	
Complacency	0.155	0.12	1.167		-0.343	0.211	0.710		0.171	0.280	1.231	
Calculation	-0.075	0.095	0.928		**0.453**	**0.191**	**1.573**		-0.288	0.240	0.750	
Collective responsibility	0.134	0.154	1.143		0.458	0.257	1.580		0.093	0.36	1.097	
**PACV**	**-0.079**	**0.017**	**0.924**	0.13	**-0.133**	**0.028**	0.876*	0.27	**-0.086**	**0.031**	**0.918**	0.18
**sub-scales**				0.15				0.33				0.30
behavior	-0.097	0.172	0.908		-0.158	0.273	0.854		0.712	0.391	2.037	
general attitude	**-0.170**	**0.045**	0.844*		**-0.309**	**0.066**	0.734*		**-0.322**	**0.100**	0.724*	
safety and efficacy	0.028	0.060	1.028		0.110	0.110	1.116		0.111	0.131	1.117	
**VCS**	**0.308**	**0.062**	**1.361**	0.15	**0.405**	**0.077**	1.500*	0.29	**0.301**	**0.113**	**1.351**	0.19
**sub-scales**				0.16				0.32				0.23
benefits	**0.202**	**0.100**	**1.224**		0.178	0.118	1.194		0.044	0.197	1.045	
harms	-0.067	0.047	0.936		-0.148	0.083	0.862		0.003	0.109	1.003	
trust	0.071	0.097	1.073		0.247	0.129	1.280		0.399	0.232	1.491	
**GVCI**	**0.731**	**0.161**	**2.076**	0.13	**1.076**	**0.206**	2.934*	0.29	**0.977**	**0.326**	**2.658**	0.22
**sub-scales**				0.15				0.30				0.30
important	0.140	0.222	1.15		**0.720**	**0.305**	**2.054**		0.948	0.621	2.580	
safe	**0.619**	**0.217**	**1.856**		-0.151	0.331	0.860		0.897	0.503	2.453	
effective	-0.087	0.254	0.916		0.126	0.337	1.134		-1.077	0.651	0.341	
compatible with religious belief	0.049	0.124	1.051		**0.373**	**0.186**	**1.453**		0.122	0.32	1.129	
**VHS**	**0.827**	**0.155**	**2.286**	0.17	**1.204**	**0.233**	3.332*	0.30	0.683	0.255	1.979	0.18
**sub-scales**				0.17				0.30				0.18
risk	-0.084	0.126	0.919		-0.149	0.206	0.862		-0.089	0.231	0.915	
lack of confidence	**0.763**	**0.192**	2.146*		**1.023**	**0.233**	2.782*		0.605	0.334	1.832	
**VAS**	**-0.485**	**0.096**	**0.616**	0.15	**-0.900**	**0.170**	0.406*	0.32	**-0.393**	**0.163**	**0.675**	0.15
**sub-scales**				0.17				0.42				0.22
safety	-0.226	0.161	0.798		-0.456	0.321	0.634		0.201	0.325	1.223	
necessity	0.130	0.183	1.139		**-1.064**	**0.341**	**0.345**		0.293	0.366	1.341	
selection & scheduling	**-0.306**	**0.152**	**0.737**		0.428	0.342	1.534		-0.531	0.326	0.588	
values/affect	0.132	0.155	1.141		-0.416	0.263	0.66		0.007	0.291	1.007	
legitimacy	-0.181	0.138	0.835		0.512	0.298	1.668		-0.274	0.273	0.76	
**VCI**	**0.778**	**0.130**	**2.178**	0.21	**0.857**	**0.168**	2.356*	0.28	**0.961**	**0.253**	2.615*	0.34
**sub-scales**				0.22				0.31				0.40
trust	0.086	0.149	1.090		-0.197	0.296	0.821		**0.985**	**0.355**	**2.678**	
importance	0.279	0.235	1.322		**0.912**	**0.326**	**2.488**		-0.464	0.469	0.629	
confidence	**0.466**	**0.222**	**1.593**		0.316	0.367	1.372		0.112	0.411	1.118	

All regressions controlled for age, gender and education levels in the second step. *R*^2^ = Nagelkerke’s *R*^2^. Bold: significant at *p* < 0.05.

* significant at < 0.001 which is the Bonferroni-corrected level of significance.

### Discussion of Study 3

In Study 3, we reached the goal of constructing a reliable and valid scale with five sub-scales, each with three items. The regression analyses showed that the 5C sub-scales are valid predictors of vaccination behavior for several vaccinations and that the amount of explained variance was relatively high. However, the analysis involved multiple tests. If we use a Bonferroni-corrected alpha level of .0015, only some remain significant as indicated in Table 7. This suggests that we should carefully interpret the results of the regressions. However, it is interesting that compared with Study 2, other sub-scales were significant predictors–e.g., while in the German elderly sample, all 5Cs were significantly related to previous flu vaccination, in the US parents sample only confidence was significantly related to flu vaccination. Contrarily, while constraints and calculation were related to MMR vaccination for US parents, for German parents only confidence played a role. Only HPV vaccination was solely predicted by confidence in both samples. Future studies should further explore country differences, potentially related to different recommendations or media coverage.

As limitations, we note a potential self-selection bias. At the beginning of the survey, it was mentioned that the survey would pose many questions about vaccination. This might have attracted people with especially high or low confidence levels, possibly accounting for confidence being an important predictor across all scales.

## General discussion

In 2015, the US National Vaccine Advisory Committee published a position paper on the current state of vaccine confidence and some of the existing approaches to increase it. It recommends the “development of an index, composed of a number of individual and social dimensions, to measure vaccine confidence. This index should be capable of (1) rapid, reliable, and valid surveillance of national vaccine confidence; (2) detection and identification of variations in vaccine confidence at the community level; and (3) diagnosis of the key dimensions that affect vaccine confidence”. We would like to expand on the concept and stress that it is important to develop such a measure that assesses not only confidence, but also other relevant factors predicting vaccination behavior. The development of the 5C scale presented here follows these extended recommendations and assesses psychological antecedents of vaccination (1) based on social and individual dimensions (e.g., by adding collective responsibility and calculation), (2) provides a validated short form to monitor hesitancy on a national and community level (Study 2), and (3) includes all aspects of vaccine hesitancy given in the literature (Table 1).

In summary, the studies showed that the pattern of significant predictors varies depending on the vaccination at hand and the target or risk group, as well as country. This is a result that holds for all existing scales. For example, in Study 2 flu vaccination was related to four factors in elderly Germans, while only one factor predicted flu vaccination in US parents. This seems to mirror the notion that “vaccine hesitancy is complex and context specific, varying across time, place and vaccines” [15].

Moreover, the analyses also showed that the general version of the 5C scale (asking for vaccination in general) predicted acceptance as similarly well as a vaccine-specific version (e.g., asking specifically for influenza vaccination). Unless the focus of a future study or intervention is only on one specific vaccination, we therefore recommend using the general scale, which can predict acceptance of several vaccinations and makes results (e.g., from different countries) easier to compare.

By relating the 5C sub-scales to psychological constructs we learned more about the psychological underpinnings of vaccine hesitancy and acceptance as described in the results of Studies 1 and 2 (summary in Table 2). If we wanted to construct ‘psychological profiles’ of the extreme ends of the scales, a person that lacks confidence is more likely to have a negative attitude and misbeliefs, mistrust the health system and medical treatments in general, and believe in conspiracies. A person who is held back due to constraints also has a more general lack of self-control and self-efficacy. Highly constrained people perceive a lack of time–so for these people vaccination should be made easy. The typical complacent person does not feel vulnerable, he or she feels healthy and does not care about the future which might lead to high-risk behaviors. Disease risks are perceived as low. People who calculate are risk averse, prefer to deliberate and are especially concerned about the risks associated with vaccination. Even though deliberation and risk assessments are important, the respective skills (numeracy) are not especially high in these people, which potentially leads to skewed risk perceptions (high vaccination risks, low disease risks). People who score high on collective responsibility generally care more for other people and are more empathic.

In summary, the analyses showed that going beyond confidence will explain vaccination behavior to a greater extent. For assessing confidence, there is a whole range of measures that all reliably and validly assess confidence (Study 3, Table 1). However, using additional concepts increased the amount of variance explained (Study 2 and Table 7).

### Limitations and future research

As a limitation of this work we have to note that the three studies, similar to the construction studies of all other existing measures, only assess concurrent validity and not predictive validity, i.e., associations with vaccination behavior that is assessed at some point in the future. This was simply beyond the scope of this work. Thus, future studies should strive to test the 5C scale’s predictive validity.

For some regressions the sample sizes were rather small (e.g., for the HPV binary logistic regression in Study 3). Additionally, for some vaccines the uptake was very high (e.g., 93% had MMR vaccination in Study 2), which reduces the variance to be explained by the scales. Future studies should strive for larger samples and therefore for more statistical power.

It is additionally important to note that the 5C scale as well as all other measures discussed in this article have been developed in WEIRD societies (Western, Educated, Industrialized, Rich, Democratic) [90]. While in developed countries, online studies may not be a problem and include people from various educational backgrounds [91], replicating such studies in developing countries may lead to an oversampling of educated participants and therefore limits the generalizability. Vaccine hesitancy is not only a problem in WEIRD societies, but also in developing, low- and middle-income countries. Thus, testing and potentially adapting the 5C scales to other contexts (e.g., such as African countries [92] or Russia [90]) is advisable. This could also include testing whether assessing religious reasons (as in the GVCI) changes the amount of explained variance in different contexts. However, it should be noted that generally vaccination is compatible with the world’s religions as analyzed by [93]. It is argued that “in multiple cases, ostensibly religious reasons to decline immunization actually reflected concerns about vaccine safety or personal beliefs among a social network of people organized around a faith community, rather than theologically based objections per se” [93]. Thus, future research should strive to disentangle these complex relations.

### 5C scale as a toolbox for diagnosis, intervention and evaluation

The 5C scale now offers a psychologically sound and validated measure to be used for regular global monitoring of the psychological antecedents of vaccination behavior. It can be used to assess the relative importance of the psychological antecedents. Knowing the relative importance is only a first step; it needs to be followed up by further exploration (e.g., in focus groups) to gain insights of potential levers of how to change the respective antecedents. For example, a lack of confidence may be related to misinformation; however, it may also be related to a political system fostering inequality; highly perceived constraints could be a function of a lack of access, inappropriate service delivery or reluctance to get registered, e.g., from a minority perspective. A broader analysis that explores the basic causes of the identified antecedents that combines qualitative and quantitative analyses are necessary, both from the perspective of the beneficiary and the provider, to develop behavior change interventions [94]. The revised “Tailoring Immunization Programmes” approach by the World Health Organization [8] uses the COM-B model to analyze behavior as a function of capability, motivation, and opportunity [95]. The 5C scale can also be used to support this work, as complacency can be interpreted as a capability aspect (knowledge, understanding importance); calculation and confidence as reflective and automatic motivation, respectively, and constraints and collective responsibility as physical and social opportunity factors. Future research should complement these efforts by identifying interventions that match the relevant C(s) [22], e.g., which interventions are best suited to overcome constraints, to increase confidence, to reduce complacency (without increasing psychological reactance), etc. Additionally, addressing more than one underlying cause in one intervention is likely to increase the success of the intervention [96]. For intervention purposes we recommend measuring the intervention’s success by comparing pre- and post-intervention data for intervention planning and evaluation.

### Conclusion

The 5C scale expands the scope of available measures and covers the broader theoretical conceptualization of vaccine hesitancy and acceptance. In contrast to other existing measures, it goes beyond capturing confidence, which proved successful in the validation studies. It can be used as a tool for diagnosis and to support the design and evaluation of clinical interventions [25]. The 5C scale allows global monitoring and comparison of the psychological antecedents of vaccination and can assist countries in collecting data to report in their annual joint reporting form [10]. There is considerable debate among practitioners and academics from all parts of the world about how to measure hesitancy right, whether hesitancy is the best term, how general such a measure can or should be, and how context-specific vaccine hesitancy is. This scale development is only one further step toward understanding vaccine hesitancy and acceptance. The greatest strength of this scale–beyond its validity–is its relation to theory and empirical association with psychological constructs. We would therefore like to offer this measure to the community for empirical testing, cultural adaption, and further development.

## Ethics statement

The studies involved human subjects and were conducted in accordance with the guidelines of the Helsinki Declaration and the German Psychological Association. They were conducted at a German University, where institutional review boards or committees are not mandatory (https://www.dgps.de/fileadmin/documents/Empfehlungen/berufsethische_richtlinien_dgps.pdf). The University of Erfurt’s IRB considered this research as exempt from the requirement of ethical approval (EV20180806). All participants gave their written informed consent to use and share their data for scientific purposes without disclosure of their identity. Analyses and data storing use anonymized data and cannot identify individual participants.

## Supporting information

S1 Table Overview of the studies. (DOCX)

S2 Table Validation constructs, their definitions and measurement (Studies 1–3). (DOCX)

S3 Table Factor loadings of the three factor analyses during the scale construction process (sorted by the final version, right). (DOCX)

S4 Table German items of the scale after Study 1. This is an intermediate product and should not be used. This intermediate version is only provided for transparency reasons. Instruction: Please evaluate how much you disagree or agree with the following statements (1 = strongly disagree, 2 = moderately disagree, 3 = neutral, 4 = moderately agree, 5 = strongly agree). Scoring: calculate mean scores of each sub-scale. Items with (R) were reverse-coded.
(DOCX)

S5 Table Concurrent and construct validity in Study 1. α = Cronbach’s alpha; bold correlations were expected. ** p < .01; *r* = Spearman-Brown correlation to examine reliability of two item measures; † the two agreeableness items correlated at .07. Therefore, only the item “I trust others easily and believe in the good in man” was selected. ^1^ assessed in sample A (*n* = 1033), ^2^ assessed in sample B (*n* = 412). SocDes = social desirability.
(DOCX)

S6 Table Convergent and discriminant validity and comparison to existing measures (Study 2). All *p*s < .001.
(DOCX)

S1 Fig Means of existing hesitancy and confidence scales (Study 3). For the means, each variable was transformed into POMP values [0,100] to allow for direct comparison of the mean values: percent of maximum possible [((observed—minimum)/(maximum—minimum)) x 100]. The PACV and the VAS were recoded for more convenient comparison. Higher values express more acceptance/positive attitudes/confidence.
(DOCX)

## Abbreviations

5A: Taxonomy for the determinants of vaccine uptake
5C: Psychological antecedents of vaccination behavior
GVCI: Global vaccine confidence index
PACV: Parental Attitudes towards Childhood Vaccination
SAGE: Strategic Advisory Group of Experts
VAS: Vaccine Acceptance Scale
VCI: Vaccine Confidence Index
VCS: Vaccine Confidence Scale
VHS: Vaccine Hesitancy Scale

## References

[1] World Health Organization. Assessment Report of the Global Vaccine Action Plan Strategic Advisory Group of Experts on Immunization. [Internet]. Geneva, Switzerland; 2017 http://www.who.int/immunization/global_vaccine_action_plan/en/

[2] Omer SB, Orenstein WA, Koplan JP. Go big and go fast—vaccine refusal and disease eradication. N Engl J Med. 2013;368: 1374–1376. 10.1056/NEJMp1300765 23574116 10.1056/NEJMp1300765

[3] Dubé E, Gagnon D, Nickels E, Jeram S, Schuster M. Mapping vaccine hesitancy—Country-specific characteristics of a global phenomenon. Vaccine. 2014;32: 6649–6654. 10.1016/j.vaccine.2014.09.039 25280436 10.1016/j.vaccine.2014.09.039 PMC5355208

[4] Larson HJ, de Figueiredo A, Xiahong Z, Schulz WS, Verger P, Johnston IG, et al The State of Vaccine Confidence 2016: Global Insights Through a 67-Country Survey. EBioMedicine. 2016;12: 295–301. 10.1016/j.ebiom.2016.08.042 27658738 10.1016/j.ebiom.2016.08.042 PMC5078590

[5] MacDonald NE, SAGE Working Group on Vaccine Hesitancy. Vaccine hesitancy: Definition, scope and determinants. Vaccine. 2015;33: 4161–4164. 10.1016/j.vaccine.2015.04.036 25896383 10.1016/j.vaccine.2015.04.036

[6] Orenstein W, Gellin B, Beigi R. Assessing the State of Vaccine Confidence in the United States: Recommendations from the National Vaccine Advisory Committee: Approved by the National Vaccine Advisory Committee on June 10, 2015. Public Health Rep. 2015;130: 573–595. 26556929 10.1177/003335491513000606 PMC4612166

[7] Betsch C, Böhm R, Chapman GB. Using behavioral insights to increase vaccination policy effectiveness. Policy Insights Behav Brain Sci. 2015;2: 61–73.

[8] Butler R, MacDonald NE, SAGE Working Group on Vaccine Hesitancy. Diagnosing the determinants of vaccine hesitancy in specific subgroups: The Guide to Tailoring Immunization Programmes (TIP). Vaccine. 2015;33: 4176–4179. 10.1016/j.vaccine.2015.04.038 25896376 10.1016/j.vaccine.2015.04.038

[9] Marti M, de Cola M, MacDonald NE, Dumolard L, Duclos P. Assessments of global drivers of vaccine hesitancy in 2014—Looking beyond safety concerns. PLOS ONE. 2017;12: e0172310 10.1371/journal.pone.0172310 28249006 10.1371/journal.pone.0172310 PMC5332020

[10] Lane S, MacDonald NE, Marti M, Dumolard L. Vaccine hesitancy around the globe: Analysis of three years of WHO/UNICEF Joint Reporting Form data-2015–2017. Vaccine. 2018; 10.1016/j.vaccine.2018.03.063 29605516 10.1016/j.vaccine.2018.03.063 PMC5999354

[11] Schmid P, Rauber D, Betsch C, Lidolt G, Denker M-L. Barriers of Influenza Vaccination Intention and Behavior–A Systematic Review of Influenza Vaccine Hesitancy, 2005–2016. PLOS ONE. 2017;12: e0170550 10.1371/journal.pone.0170550 28125629 10.1371/journal.pone.0170550 PMC5268454

[12] Brewer NT, Chapman GB, Rothman AJ, Leask J, Kempe A. Increasing Vaccination: Putting Psychological Science Into Action: Psychol Sci Public Interest. 2018; 10.1177/1529100618760521 29611455 10.1177/1529100618760521

[13] Larson HJ, Jarrett C, Eckersberger E, Smith DMD, Paterson P. Understanding vaccine hesitancy around vaccines and vaccination from a global perspective: A systematic review of published literature, 2007–2012. Vaccine. 2014;32: 2150–2159. 10.1016/j.vaccine.2014.01.081 24598724 10.1016/j.vaccine.2014.01.081

[14] Thomson A, Robinson K, Vallée-Tourangeau G. The 5As: A practical taxonomy for the determinants of vaccine uptake. Vaccine. 2016;34: 1018–1024. 10.1016/j.vaccine.2015.11.065 26672676 10.1016/j.vaccine.2015.11.065

[15] The SAGE Vaccine Hesitancy Working Group. Report of the SAGE working group on vaccine hesitancy. 2014; http://www.who.int/immunization/sage/sage_wg_vaccine_hesitancy_apr12/en/index.html

[16] European Commission. Report on the Conference on childhood immunisation: progress, challenges and priorities for further action; 2012; https://ec.europa.eu/health/sites/health/files/vaccination/docs/ev_20121016_mi_en.pdf

[17] Opel DJ, Taylor JA, Zhou C, Catz S, Myaing M, Mangione-Smith R. The relationship between parent attitudes about childhood vaccines survey scores and future child immunization status: a validation study. JAMA Pediatr. 2013;167: 1065–1071. 10.1001/jamapediatrics.2013.2483 24061681 10.1001/jamapediatrics.2013.2483 PMC4957941

[18] Gilkey MB, Reiter PL, Magnus BE, McRee A-L, Dempsey AF, Brewer NT. Validation of the vaccination confidence scale: a brief measure to identify parents at risk for refusing adolescent vaccines. Acad Pediatr. 2016;16: 42–49. 10.1016/j.acap.2015.06.007 26300368 10.1016/j.acap.2015.06.007 PMC4715593

[19] Shapiro GK, Holding A, Perez S, Amsel R, Rosberger Z. Validation of the vaccine conspiracy beliefs scale. Papillomavirus Res. 2016;2: 167–172. 10.1016/j.pvr.2016.09.001 29074176 10.1016/j.pvr.2016.09.001 PMC5886898

[20] Sarathchandra D, Navin MC, Largent MA, McCright AM. A survey instrument for measuring vaccine acceptance. Prev Med. 2018;109: 1–7. 10.1016/j.ypmed.2018.01.006 29337069 10.1016/j.ypmed.2018.01.006

[21] Frew PM, Murden R, Mehta C, Chamberlain A, Hinman A, Nowak G, et al. Development of an Index for Measurement of Parents’ Vaccine Confidence and Linkage to Pediatric Immunization Acceptance; undated; https://www.hhs.gov/sites/default/files/Frew_Development%20of%20a%20Vaccine%20Confidence%20Index%20to%20Measure%20Parental%20Confidence%20in%20Childhood%20Vaccinations_remediated.pdf

[22] Betsch C, Böhm R, Chapman GB. Using behavioral insights to increase vaccination policy effectiveness. Policy Insights Behav Brain Sci. 2015;2: 61–73.

[23] Ajzen I. The theory of planned behavior. Organ Behav Hum Decis Process. 1991;50: 179–211.

[24] Carpenter CJ. A meta-analysis of the effectiveness of health belief model variables in predicting behavior. Health Commun. 2010;25: 661–669. 10.1080/10410236.2010.521906 21153982 10.1080/10410236.2010.521906

[25] Betsch C, Rossmann C, Pletz MW, Vollmar HC, Freytag A, Wichmann O, et al Increasing influenza and pneumococcal vaccine uptake in the elderly: Study protocol for the multi-methods prospective intervention study Vaccination60+. BMC Public Health. subm. for publication; 10.1186/s12889-018-5787-9 PMC6048840 30012141

[26] Stanovich KE, West RF. Individual differences in reasoning: Implications for the rationality debate? Behav Brain Sci. 2000;23: 645–665. 10.1017/S0140525X00003435 11301544 10.1017/s0140525x00003435

[27] Fine P, Eames K, Heymann DL. “Herd Immunity”: A Rough Guide. Clin Infect Dis. 2011;52: 911–916. 10.1093/cid/cir007 21427399 10.1093/cid/cir007

[28] Bedford H, Attwell K, Danchin M, Marshall H, Corben P, Leask J. Vaccine hesitancy, refusal and access barriers: The need for clarity in terminology. Vaccine. 2017; 10.1016/j.vaccine.2017.08.004 28830694 10.1016/j.vaccine.2017.08.004

[29] Gilkey MB, Magnus BE, Reiter PL, McRee A-L, Dempsey AF, Brewer NT. The Vaccination Confidence Scale: a brief measure of parents’ vaccination beliefs. Vaccine. 2014;32: 6259–6265. 10.1016/j.vaccine.2014.09.007 25258098 10.1016/j.vaccine.2014.09.007 PMC4418546

[30] Larson HJ, Jarrett C, Schulz WS, Chaudhuri M, Zhou Y, Dube E, et al Measuring vaccine hesitancy: the development of a survey tool. Vaccine. 2015;33: 4165–4175. 10.1016/j.vaccine.2015.04.037 25896384 10.1016/j.vaccine.2015.04.037

[31] Shapiro GK, Tatar O, Dube E, Amsel R, Knauper B, Naz A, et al The vaccine hesitancy scale: Psychometric properties and validation. Vaccine. 2018;36: 660–667. 10.1016/j.vaccine.2017.12.043 29289384 10.1016/j.vaccine.2017.12.043

[32] Askelson NM, Campo S, Lowe JB, Smith S, Dennis LK, Andsager J. Using the theory of planned behavior to predict mothers’ intentions to vaccinate their daughters against HPV. J Sch Nurs. 2010;26: 194–202. 10.1177/1059840510366022 20335232 10.1177/1059840510366022

[33] Zingg A, Siegrist M. Measuring people’s knowledge about vaccination: developing a one-dimensional scale. Vaccine. 2012;30: 3771–3777. 10.1016/j.vaccine.2012.03.014 22445808 10.1016/j.vaccine.2012.03.014

[34] Egede LE, Ellis C. Development and testing of the multidimensional trust in health care systems scale. J Gen Intern Med. 2008;23: 808 10.1007/s11606-008-0613-1 18415653 10.1007/s11606-008-0613-1 PMC2517872

[35] Horne R, Weinman J, Hankins M. The beliefs about medicines questionnaire: The development and evaluation of a new method for assessing the cognitive representation of medication. Psychol Health. 1999;14: 1–24. 10.1080/08870449908407311

[36] Bruder M, Haffke P, Neave N, Nouripanah N, Imhoff R. Measuring individual differences in generic beliefs in conspiracy theories across cultures: Conspiracy Mentality Questionnaire. Front Psychol. 2013;4: 225 10.3389/fpsyg.2013.00225 23641227 10.3389/fpsyg.2013.00225 PMC3639408

[37] Schwarzer R, Fuchs R. Self-efficacy and health behaviours. Predict Health Behav Res Pract Soc Cogn Models. 1996; 163–196.

[38] Fischer P, Kastenmüller A, Greitemeyer T, Fischer J, Frey D, Crelley D. Threat and selective exposure: the moderating role of threat and decision context on confirmatory information search after decisions. J Exp Psychol Gen. 2011;140: 51–62. 10.1037/a0021595 21171802 10.1037/a0021595

[39] Askelson NM, Campo S, Lowe JB, Smith S, Dennis LK, Andsager J. Using the theory of planned behavior to predict mothers’ intentions to vaccinate their daughters against HPV. J Sch Nurs. 2010;26: 194–202. 10.1177/1059840510366022 20335232 10.1177/1059840510366022

[40] Brewer NT, Cuite CL, Herrington JE, Weinstein ND. Risk compensation and vaccination: Can getting vaccinated cause people to engage in risky behaviors? Ann Behav Med. 2007;34: 95–99. 17688401 10.1007/BF02879925

[41] Petrocelli JV. Factor validation of the Consideration of Future Consequences Scale: evidence for a short version. J Soc Psychol. 2003;143: 405–413. 10.1080/00224540309598453 12934832 10.1080/00224540309598453

[42] Johnson JG, Wilke A, Weber EU. DOSPERT-G Bereichsspezifische Risikoskala–Deutsche Version Domain-specific Risk-taking Scale–German version; 2004; http://citeseerx.ist.psu.edu/viewdoc/download;jsessionid=A4AE81167DD98377ED644D86F23F6946?doi=10.1.1.372.4920&rep=rep1&type=pdf

[43] Lapsley DK, Hill PL. Subjective invulnerability, optimism bias and adjustment in emerging adulthood. J Youth Adolesc. 2010;39: 847–857. 10.1007/s10964-009-9409-9 20596815 10.1007/s10964-009-9409-9

[44] Luszczynska A, Scholz U, Schwarzer R. The General Self-Efficacy Scale: Multicultural Validation Studies. J Psychol. 2005;139: 439–457. 10.3200/JRLP.139.5.439-457 16285214 10.3200/JRLP.139.5.439-457

[45] Fadda M, Galimberti E, RomanÃ L, Faccini M, Senatore S, Zanetti A, et al Validation of a scale to measure parental psychological empowerment in the vaccination decision. J Public Health Res. 2017;6 10.4081/jphr.2017.955 29071257 10.4081/jphr.2017.955 PMC5641652

[46] Holm EJ, Holroyd K. The Daily Hassles Scale (Revised): Does it measure stress or symptoms? Behav Assess. 1992;14: 465–482.

[47] Cylus J, Papanicolas I. An analysis of perceived access to health care in Europe: How universal is universal coverage? Health Policy. 2015;119: 1133–1144. 10.1016/j.healthpol.2015.07.004 26252959 10.1016/j.healthpol.2015.07.004

[48] Kata A. Anti-vaccine activists, Web 2.0, and the postmodern paradigm–An overview of tactics and tropes used online by the anti-vaccination movement. Vaccine. 2012;30: 3778–3789. 10.1016/j.vaccine.2011.11.112 22172504 10.1016/j.vaccine.2011.11.112

[49] Dubé E, Laberge C, Guay M, Bramadat P, Roy R, Bettinger JA. Vaccine hesitancy: An overview. Hum Vaccines Immunother. 2013;9: 1763–1773. 10.4161/hv.24657 23584253 10.4161/hv.24657 PMC3906279

[50] Dixon G, Clarke C. The effect of falsely balanced reporting of the autism-vaccine controversy on vaccine safety perceptions and behavioral intentions. Health Educ Res. 2013;28: 352–359. 10.1093/her/cys110 23193194 10.1093/her/cys110

[51] Betsch C. Präferenz für Intuition und Deliberation (PID). Z Für Differ Diagn Psychol. 2004;25: 179–197. 10.1024/0170-1789.25.4.179

[52] Wiseman R, Watt C. Measuring superstitious belief: Why lucky charms matter. Personal Individ Differ. 2004;37: 1533–1541.

[53] Cokely ET, Galesic M, Schulz E, Ghazal S, Garcia-Retamero R. Measuring risk literacy: The Berlin Numeracy Test. Judgm Decis Mak. 2012;7: 25–47.

[54] Betsch C, Böhm R, Korn L. Inviting free-riders or appealing to prosocial behavior? Game-theoretical reflections on communicating herd immunity in vaccine advocacy. Health Psychol. 2013;32: 978–985. 10.1037/a0031590 24001248 10.1037/a0031590

[55] Betsch C, Böhm R, Korn L, Holtmann C. On the benefits of explaining herd immunity in vaccine advocacy. Nat Hum Behav. 2017; 10.1038/s41562-017-0056

[56] Shulruf B, Hattie J, Dixon R. Development of a new measurement tool for individualism and collectivism. J Psychoeduc Assess. 2007;25: 385–401.

[57] Clark MS, Oullette R, Powell MC, Milberg S. Recipient’s mood, relationship type, and helping. J Pers Soc Psychol. 1987;53: 94 3612495 10.1037//0022-3514.53.1.94

[58] Spreng RN, McKinnon MC, Mar RA, Levine B. The Toronto Empathy Questionnaire: Scale development and initial validation of a factor-analytic solution to multiple empathy measures. J Pers Assess. 2009;91: 62–71. 10.1080/00223890802484381 19085285 10.1080/00223890802484381 PMC2775495

[59] Simms LJ. Classical and Modern Methods of Psychological Scale Construction. Soc Personal Psychol Compass. 2008;2: 414–433. 10.1111/j.1751-9004.2007.00044.x

[60] Greiner B. The Online Recruitment System ORSEE—A Guide for the Organization of Experiments in Economics [Internet]. Max Planck Institute of Economics, Strategic Interaction Group; 2004. https://EconPapers.repec.org/RePEc:esi:discus:2003-10

[61] Clark LA, Watson D. Constructing validity: Basic issues in objective scale development. Psychol Assess. 1995;7: 309–319. 10.1037/1040-3590.7.3.309

[62] Faul F, Erdfelder E, Buchner A, Lang A-G. Statistical power analyses using G*Power 3.1: Tests for correlation and regression analyses. Behav Res Methods. 2009;41: 1149–1160. 10.3758/BRM.41.4.1149 19897823 10.3758/BRM.41.4.1149

[63] Ajzen I. Constructing a Theory of Planned Behavior Questionnaire. 2006; 1–12. https://www.researchgate.net/publication/235913732_Constructing_a_Theory_of_Planned_Behavior_Questionnaire

[64] Cokely ET, Galesic M, Schulz E, Ghazal S, Garcia-Retamero R. Measuring Risk Literacy: The Berlin Numeracy Test. Judgm Decis Mak. 2012;7: 25–47.

[65] Betsch C, Haase N, Renkewitz F, Schmid P. The narrative bias revisited: What drives the biasing influence of narrative information on risk perceptions? Judgm Decis Mak. 2015;10: 241.

[66] Kemper CJ, Beierlein C, Bensch D, Kovaleva A, Rammstedt B. Eine Kurzskala zur Erfassung des Gamma-Faktors sozial erwünschten Antwortverhaltens: die Kurzskala Soziale Erwünschtheit-Gamma (KSE-G). 2012;2012/25. https://www.ssoar.info/ssoar/bitstream/handle/document/33958/ssoar-2012-kemper_et_al-Eine_Kurzskala_zur_Erfassung_des.pdf?sequence=1

[67] Rammstedt B, Kemper C, Klein M, Beierlein C, Kovaleva A. Eine kurze Skala zur Messung der fünf Dimensionen der Persönlichkeit: Big-Five-Inventory-10 (BFI-10) [A Short Scale for Assessing the Big Five Dimensions of Personality—10 Item Big Five Inventory (BFI-10)]. Methoden–Daten–Anal. 2013;7: 233–249.

[68] Preacher KJ, MacCallum RC. Repairing Tom Swift’s Electric Factor Analysis Machine. Underst Stat. 2003;2: 13–43. 10.1207/S15328031US0201_02

[69] Bauch CT, Earn DJD. Vaccination and the theory of games. Proc Natl Acad Sci U S A. 2004;101: 13391–13394. 10.1073/pnas.0403823101 15329411 10.1073/pnas.0403823101 PMC516577

[70] Chapman GB, Li M, Vietri J, Ibuka Y, Thomas D, Yoon H, et al Using game theory to examine incentives in influenza vaccination behavior. Psychol Sci. 2012;23: 1008–1015. 10.1177/0956797612437606 22810166 10.1177/0956797612437606

[71] Quadri-Sheriff M, Hendrix KS, Downs SM, Sturm LA, Zimet GD, Finnell SME. The Role of Herd Immunity in Parents’ Decision to Vaccinate Children: A Systematic Review. PEDIATRICS. 2012;130: 522–530. 10.1542/peds.2012-0140 22926181 10.1542/peds.2012-0140

[72] Böhm R, Betsch C, Korn L. Selfish-rational non-vaccination: Experimental evidence from an interactive vaccination game. J Econ Behav Organ. 2016;131: 183–195. 10.1016/j.jebo.2015.11.008

[73] Strelitz B, Gritton J, Klein EJ, Bradford MC, Follmer K, Zerr DM, et al Parental vaccine hesitancy and acceptance of seasonal influenza vaccine in the pediatric emergency department. Vaccine. 2015;33: 1802–1807. 10.1016/j.vaccine.2015.02.034 25744225 10.1016/j.vaccine.2015.02.034 PMC7864559

[74] Williams SE, Morgan A, Opel D, Edwards K, Weinberg S, Rothman R. Screening Tool Predicts Future Underimmunization Among a Pediatric Practice in Tennessee. Clin Pediatr (Phila). 2016;55: 537–542. 10.1177/0009922815615823 26581360 10.1177/0009922815615823 PMC7864550

[75] Glanz JM, Wagner NM, Narwaney KJ, Kraus CR, Shoup JA, Xu S, et al Web-based Social Media Intervention to Increase Vaccine Acceptance: A Randomized Controlled Trial. Pediatrics. 2017;140 10.1542/peds.2017-1117 29109107 10.1542/peds.2017-1117 PMC8574135

[76] Henrikson NB, Opel DJ, Grothaus L, Nelson J, Scrol A, Dunn J, et al Physician Communication Training and Parental Vaccine Hesitancy: A Randomized Trial. PEDIATRICS. 2015;136: 70–79. 10.1542/peds.2014-3199 26034240 10.1542/peds.2014-3199

[77] Williams SE, Rothman RL, Offit PA, Schaffner W, Sullivan M, Edwards KM. A randomized trial to increase acceptance of childhood vaccines by vaccine-hesitant parents: a pilot study. Acad Pediatr. 2013;13: 475–480. 10.1016/j.acap.2013.03.011 24011750 10.1016/j.acap.2013.03.011 PMC3767934

[78] Amin AB, Bednarczyk RA, Ray CE, Melchiori KJ, Graham J, Huntsinger JR, et al Association of moral values with vaccine hesitancy. Nat Hum Behav. 2017;1: 873–880. 10.1038/s41562-017-0256-5 10.1038/s41562-017-0256-5 31024188

[79] Napolitano F, D’Alessandro A, Angelillo IF. Investigating Italian parents’ vaccine hesitancy: A cross-sectional survey. Hum Vaccines Immunother. 2018; 14(7):1558–1565. 10.1080/21645515.2018.1463943 29641945 10.1080/21645515.2018.1463943 PMC6067864

[80] Mohd Azizi FS, Kew Y, Moy FM. Vaccine hesitancy among parents in a multi-ethnic country, Malaysia. Vaccine. 2017;35: 2955–2961. 10.1016/j.vaccine.2017.04.010 28434687 10.1016/j.vaccine.2017.04.010

[81] McRee A-L, Brewer NT, Reiter PL, Gottlieb SL, Smith JS. The Carolina HPV immunization attitudes and beliefs scale (CHIAS): scale development and associations with intentions to vaccinate. Sex Transm Dis. 2010;37: 234–239. 10.1097/OLQ.0b013e3181c37e15 19940807 10.1097/OLQ.0b013e3181c37e15

[82] Gilkey MB, McRee A-L, Magnus BE, Reiter PL, Dempsey AF, Brewer NT. Vaccination Confidence and Parental Refusal/Delay of Early Childhood Vaccines. Moore AC, editor. PLOS ONE. 2016;11: e0159087 10.1371/journal.pone.0159087 27391098 10.1371/journal.pone.0159087 PMC4938536

[83] Hays RD, Morales LS. The RAND-36 measure of health-related quality of life. Ann Med. 2001;33: 350–357. 11491194 10.3109/07853890109002089

[84] Tangney JP, Baumeister RF, Boone AL. High Self-Control Predicts Good Adjustment, Less Pathology, Better Grades, and Interpersonal Success. J Pers. 2004;72: 271–324. 10.1111/j.0022-3506.2004.00263.x 15016066 10.1111/j.0022-3506.2004.00263.x

[85] Bundeszentrale für gesundheitliche Aufklärung. Impfkalender 2017/2018—Welche Impfungen sind empfohlen? Empfehlungen der Ständigen Impfkommission (STIKO), Stand: August 2017. 2017; https://www.impfen-info.de/fileadmin/impfen-info.de/Downloads/Impfkalender_2017-2018.pdf

[86] Berinsky AJ, Margolis MF, Sances MW. Separating the Shirkers from the Workers? Making Sure Respondents Pay Attention on Self-Administered Surveys. Am J Polit Sci. 2014;58: 739–753. 10.1111/ajps.12081

[87] Perez S, Shapiro GK, Tatar O, Joyal-Desmarais K, Rosberger Z. Development and Validation of the Human Papillomavirus Attitudes and Beliefs Scale in a National Canadian Sample: Sex Transm Dis. 2016;43: 626–632. 27631357 10.1097/OLQ.0000000000000506

[88] Moeller J. A word on standardization in longitudinal studies: don’t. Front Psychol. 2015;6 10.3389/fpsyg.2015.01389 26441764 10.3389/fpsyg.2015.01389 PMC4569815

[89] Centers for Disease Control and Prevention (CDC). Immunization Schedules | CDC. 2018. https://www.cdc.gov/vaccines/schedules/index.html

[90] Henrich J, Heine SJ, Norenzayan A. The weirdest people in the world? Behav Brain Sci. 2010;33: 61–83. 10.1017/S0140525X0999152X 20550733 10.1017/S0140525X0999152X

[91] Bartneck C, Duenser A, Moltchanova E, Zawieska K. Comparing the Similarity of Responses Received from Studies in Amazon’s Mechanical Turk to Studies Conducted Online and with Direct Recruitment. Voracek M, editor. PLOS ONE. 2015;10: e0121595 10.1371/journal.pone.0121595 25876027 10.1371/journal.pone.0121595 PMC4397064

[92] Cooper S, Betsch C, Sambala EZ, Mchiza N, Wiysonge CS. Vaccine hesitancy–a potential threat to the achievements of vaccination programmes in Africa. Hum Vaccines Immunother. 2018; 00–00. 10.1080/21645515.2018.1460987 29617173 10.1080/21645515.2018.1460987 PMC6284499

[93] Grabenstein JD. What the World’s religions teach, applied to vaccines and immune globulins. Vaccine. 2013;31: 2011–2023. 10.1016/j.vaccine.2013.02.026 23499565 10.1016/j.vaccine.2013.02.026

[94] Rossmann C. Strategic Health Communication. Theory- and Evidence-Based Campaign Development In: Holtzhausen D, Zerfass A, editors. The Routledge Handbook of Strategic Communication. New York; London: Routledge/Taylor & Francis Group; pp. 409–423.

[95] Michie S, van Stralen MM, West R. The behaviour change wheel: A new method for characterising and designing behaviour change interventions. Implement Sci. 2011;6 10.1186/1748-5908-6-42 21513547 10.1186/1748-5908-6-42 PMC3096582

[96] Frew PM, Lutz CS. Interventions to increase pediatric vaccine uptake: An overview of recent findings. Hum Vaccines Immunother. 2017;13: 2503–2511. 10.1080/21645515.2017.1367069 28949819 10.1080/21645515.2017.1367069 PMC5703404

