# Supplementary material for: Beyond confidence: Development of a measure assessing the 5C psychological antecedents of vaccination
Source: PLoS One. 2018 Dec 7;13(12):e0208601. doi: 10.1371/journal.pone.0208601 (PMC6285469; doi:10.1371/journal.pone.0208601)
Supplement: S1 Table — (DOCX) [file pone.0208601.s001.docx]

**S1 Table**

|  | Study 1  (Supplement) | Study 2 | Study 3 |
| --- | --- | --- | --- |
| Sample | *Online convenience sample*  *N* = 1,033  *M*_age_ = 32.92, *SD* = 9.37, 71% female; 495 were parents of at least one child  *Student convenience sample*  *N* = 412  *M*_age_ = 22.21, *SD* = 3.55, 83% female | *Nationally representative sample*  *N* = 1,003 for age, gender and parenthood  *M_age_* = 47.98 (*SD* = 15.62), 51% were female; 15% were parents of a child between 11 months and 6 years of age, 16% had girls between 9 and 17 years of age, 31% of the participants were over 60 years of age, 29% had traveled in risk areas | *Online sample*  *N* = 350  *M*_age_ = 34.01, *SD* = 7.49; 49% female, 92% were parents of a child above 2 years, 28% had children above 11 years of age |
| Aim | Item generation and selection | Refinement, validation and comparison to existing measures  Short version | Refinement of collective responsibility scale |
