# Supplementary material for: Beyond confidence: Development of a measure assessing the 5C psychological antecedents of vaccination
Source: PLoS One. 2018 Dec 7;13(12):e0208601. doi: 10.1371/journal.pone.0208601 (PMC6285469; doi:10.1371/journal.pone.0208601)
Supplement: S2 Table — (DOCX) [file pone.0208601.s002.docx]

**S2 Table**

| **Construct** | **Definition** | **Measure and Reference** |
| --- | --- | --- |
| **Study 1** |  |  |
| **CONFIDENCE** |  |  |
| Attitude | “Attitude is the degree to which performance of the behavior is positively or negatively valued.” [1] | Scale: 3 items (e.g., “Vaccination is necessary.”)[2]  Questions measuring attitudes are based on questions from [1]  Response option: 5-point scale (1 = strongly disagree, 5 = strongly agree)  Score: A mean of the sum of these three responses is calculated. |
| Beliefs about medicines | The construct is defined as the cognitive representation of medication. It describes the way people see medicines, e.g., as harmful and overused by doctors. The beliefs correlate with the reported adherence to treatments – those who believe that medicines are overused by doctors report lower medication adherence rates [3]. | Scale: 7 items (e.g., “Medicines do more harm than good.”)[3]  Response option: 5-point scale (1 = strongly disagree, 5 = strongly agree).  Score: A mean score of all items is calculated. |
| Conspiracy mentality | Conspiracy mentality can be described as “a general susceptibility to explanations based on conspiracy theories”. The instrument “assesses differences in generic tendency to engage in conspiracist ideation within and across cultures” [4]. | Scale: 5 items (e.g., “I think … many very important things happen in the world, which the public are never informed about.”)[4]  Response option: 11-point scale  (0% = certainly not, 100% = certain). |
| **COMPLACENCY** |  |  |
| Risk attitude | “Risk attitude is … a descriptive label for the shape of the utility function presumed to  underlie a person’s choices. It describes the shape of his or her utility function (derived  from a series of risky choices) for the outcomes in question.” [5]. | Scale: 8 items, sub-scale health-related behaviors (e.g., “How likely is it that you are going to buy an illegal drug?”) taken from the domain-specific Risk-taking Scale – German version (DOSPERT-G). [6]  Response option: 5-point scale (1 = very unlikely, 5 = very likely).  Score: A mean of the sum of these responses is calculated. |
| Consideration of future consequences | The construct is defined as the extent to which individuals consider distant versus immediate consequences of potential behaviors [7]. | Scale: 12 items (e.g., “I consider how things might be in the future and try to influence those things with my day to day behavior.”)  Response option: 5-point scale (1 = extremely uncharacteristic, 5 = extremely characteristic).  Score: A mean of the sum of these responses is calculated. |
| Perceived risk of disease | See definition of risk perception:  Defined as the likelihood and magnitude of potential infection (including non-health costs and benefits) [8]. | Scale: 1 item (“How risky do you judge [name of disease] to be, if you do not get vaccinated?”)  Response option: 100-point scale (1 = not risky, 100 = absolutely risky). |
| Perceived risk of vaccination | See definition of risk perception:  Defined as the likelihood and magnitude of potential vaccine side effects (including non-health costs and benefits) [8]. | Scale: 1 item (“How risky do you judge the vaccine against [name of disease] to be?”)  Response option: 100-point scale (1 = not risky, 100 = absolutely risky). |
| **CONSTRAINTS** |  |  |
| Empowerment | Psychological health empowerment is defined as “the belief and claim that it is within reach of a person to contribute substantially to protect and regain his or her own health” | Scale: 4 items (e.g., “My decision about my child’s vaccinations is especially driven by my personal experiences with vaccinations and diseases.”) [9]  Response option: 6-point scale (1 = absolutely disagree, 6 = absolutely agree). |
| Perceived behavioral control | Perceived behavioral control (PBC) is defined as individual control beliefs regarding the performance of a behavior [1]. In this case, the perceived ease or difficulty of getting vaccinated. | Scale: 4 items (e.g., “It is mostly up to me whether I get vaccinated.”)  [2]  Response option: 7-point scale (1 = I totally disagree, 7 = I totally agree).  Score: Individual PBC score is calculated as the mean of all items. |
| Self-efficacy | Self-efficacy (SE) is defined as “people’s beliefs about their capabilities to exercise control over their own level of functioning and over events that affect their lives” [10] (p. 257). | Scale: 10 items (e.g., “Thanks to my resourcefulness, I can handle unforeseen situations”).  [11]  Response option: 4-point scale (1 = not at all true, 7 = exactly true).  Score: Individual SE score is calculated as the mean of all items. |
| **CALCULATION** |  |  |
| Numeracy | “Numeracy is defined as aptitude with probabilities, fractions, and ratios” [12]. | Scale: 1 item (“Imagine that we roll a 6-sided die 50 times. Out of 50 rolls, how many times do you think the die would come up uneven (1,3 or 5)?”)  (taken from [12], item originally developed by [13])  Response option: open-ended response.  Score: dichotomized item responses to be either correct or incorrect - scored 1 and 0. |
| Subjective norms | “Subjective norms are what important referent groups want an individual to do and individual’s willingness to comply with these groups” [2]. In this case, subjective norms are characterized by the perceived pressure to engage in vaccination behavior. | Scale: 4 items (e.g., “Most people think I should vaccinate.”) [2]  Response option: 7-point scale (1 = I totally disagree, 7 = I totally agree).  Score: Individual norm score is calculated as a mean of all items. |
| **Explorative** |  |  |
| Big 5 | Taxonomy of personality. Assumes that personality is ordered hierarchically from a large number of specific traits to a much smaller number of general characteristics [14–16]. | Scale: 10 items. 2 items for each personality dimension (e.g., “I see myself as someone who is reserved.”) [17]  Response option: 5-point scale (1 = disagree strongly, 5 = agree strongly).  Score: A mean of the sum of these responses per facet is calculated. |
| Social desirability | Social desirability is defined as the tendency of an individual to give overly positive self-descriptions of themselves [18] and can be deconstructed into two dimensions: minimizing of negative qualities and exaggerating of positive qualities. | Scale: 4 items (e.g., “Even if I am feeling stressed, I am always friendly and polite to others.”)  [19]  Response option: 5-point scale (1 = doesn't apply at all, 5 = applies completely).  Score: A mean of the sum of these responses is calculated. |

| **Study 2** |  |  |
| --- | --- | --- |
| **CONFIDENCE** |  |  |
| Attitude | “Attitude is the degree to which performance of the behavior is positively or negatively valued” [1] | Scale: 3 items (e.g., “Vaccination is necessary.”) [2]  Questions measuring attitudes are based on questions from [1].  Response option: 5-point scale (1 = strongly disagree, 5 = strongly agree).  Score: A mean of the sum of these three responses is calculated. |
| Vaccination knowledge scale | Vaccination knowledge is defined as a set of items that reflects common misconceptions about vaccinations in general including “questions about the immunization process related to vaccination, the impact of vaccination, and the consequences of vaccination” [20]. | Scale: 9 items (e.g., “The efficacy of vaccines has been proven.”) [20]  Response option: correct, incorrect, do not know.  Score: Knowledge items are recoded into dichotomous variables for data analysis (1 = correct answer, 0 = incorrect answer and do not know). A total score is calculated as a sum of all correct answers. |
| Trust in health care systems (provider, payer, institution) | Trust in health care systems can be defined as how patients perceive health care providers, payers and institutions in terms of their competence, honesty, confidentiality and fidelity/agency [21]. | Scale: 17 items, 3 sub-scales  Trust in health care providers (10 items, e.g., “My health care provider is usually considerate of my needs and puts them first.”), trust in health care payers (4 items, e.g., “Health care payers are good at what they do.”), trust in health care institutions (3 items, e.g., “Health care institutions provide the highest quality in medical care.”) [21]  Response option: 5-point scale (1 = strongly disagree, 5 = strongly agree).  Score: “A summary score consisting of the sum of individual items was created, so that higher total and sub-scale scores represented greater trust in health care systems.” |
| Conspiracy mentality | Conspiracy mentality can be described as “a general susceptibility to explanations based on conspiracy theories.” The instrument “assesses differences in generic tendency to engage in conspiracist ideation within and across cultures” [4]. | Scale: 5 items (e.g., “I think … many very important things happen in the world, which the public is never informed about.”) [4]  Response option: 11-point scale (0% = certainly not, 100% = certain). |
| **CALCULATION** |  |  |
| Superstitious belief | “Superstitions reflect the notion that certain behaviors (e.g., breaking a mirror) or omens (e.g., seeing a black cat) are magically associated with unlucky and potentially harmful consequences” [22]. | Scale: 6 items  Negative superstitions (3 items, e.g., “Have you avoided walking under a ladder because it is associated with bad luck?”)  Positive superstitions (3 items, e.g., “Do you say “fingers crossed” or actually cross your fingers?”) [22]  Response option: 5-point scale (1 = definitely no, 5 = definitely yes)  Score: The positive and negative items are summed separately to provide a measure for the degree with which participants endorse these superstitions. |
| Preference for Intuition and Deliberation (PID) | Intuition is defined as “a basic mode that uses direct affective reactions towards the decision option as the decision criterion (affect-based decision making)”.  Deliberation is defined as “a reflective, cognition-based mode (beliefs, evaluations, reasons)” [23,24] | Scale: 18 items, 2 sub-scales Sub-scale Preference for intuition, PID-Intuition (9 items, e.g., “My feelings play an important role in my decisions.”)  Sub-scale Preference for deliberation, PID-Deliberation (9 items, e.g., “I prefer making detailed plans rather than leaving things to chance.”)[23]  Response option: 5-point scale (1 = strongly disagree, 5 = strongly agree) |
| **COMPLACENCY** |  |  |
| Perceived threat of disease |  | Scale: 1 item (“Suppose you do not have measles-mumps-rubella vaccination and you are not yet suffering from measles: How high do you estimate the likelihood of measles in your lifetime?”)  Scale: 1 item, 110-point scale (1 = 0%, 10 = 100%). |
| Perceived health status | The perceived health status reflects an individual's awareness of symptoms, diagnoses, and performance decrements that are associated with mortality risk [25]. | Scale: 4 items (e.g., “I expect my health to get worse.”) taken from General health perceptions of the RAND 36 [26]  Response option: 5-point scale (1 = definitely false, 5 definitely true)  and  5-point scale: “In general, would you say your health is…” (1 = poor, 5 = excellent). |
| Invulnerability | Invulnerability is defined as a felt sense of invulnerability to injury, harm and danger [27].  Is defined as an individual’s sense of personal indestructibility in the face of physical and psychological risks [28]. | Scale: 20 items (e.g., “I’m unlikely to get hurt if I do a dangerous thing.”), taken from Adolescent Invulnerability Scale (AIS). [27]  Response option: 5-point scale (1 = strongly disagree, 5 = strongly agree).  Scoring: A total score is tallied across the 20 items, with high scores representing a greater proclivity for delinquent behavior. |
| **CONSTRAINTS** |  |  |
| Empowerment | Psychological health empowerment is defined as “the belief and claim that it is within reach of a person to contribute substantially to protect and regain his or her own health” [9]. | Scale: 4 items (e.g., “My decision about my child’s vaccinations is especially driven by my personal experiences with vaccinations and diseases.”) [9]  Response option: 6-point scale (1 = absolutely disagree, 6 = absolutely agree). |
| Self-control | Self-control (SC) is described as “the ability to regulate the self strategically in response to goals, priorities, and environmental demands” [29]. | Scale: 36 items, 5 sub-scales (self-discipline, non-impulsive action, healthy habits, regulation in service of a work ethic, reliability) (e.g., “I have a hard time breaking bad habits.”) [29]  Response option: 5-point scale (1 = not at all like me, 5 = very much like me).  Score: An individual SC score is calculated as the mean of all items. |
| Perceived time pressure | Perceived time pressure is a daily hassle that can cause time stress [30]. Time pressure arises when current demands of time exceed available resources. | Scale: 11 items (e.g., “Too many things to do, too many interruptions.”), taken from the daily hassle scale related to time pressure.  First introduced by [31] and revised by [30].  Response option: Participants indicated occurrence and severity on a 4-point scale (0 = did not occur to me, 3 = the hassle was extremely severe).  Scores: Individual perceived TP is calculated as two scores: frequency (how many hassles) and intensity (sum score of severity divided by number of hassles). |
| Perceived access to health care | Perceived access to health care is the individual perceived likelihood of getting necessary health care if needed. | Scale: 1 item (“Please report the likelihood of accessing health care if you should need it in the next 12 months?”) [32]  Response option: 4-point scale (1 = not at all likely, 4 = very likely).  Score: Individual SC score is calculated based on one item. |
| **COLLECTIVE RESPONSIBILITY** |  |  |
| Communal Orientation | In communal relationships, people presumably feel responsible for others’ welfare. They desire and/or feel obligated to benefit another person when he or she has a need. They may also benefit the other person simply to please and to show a general concern for his or her welfare. In addition, they expect the other person to be responsive to their needs and to demonstrate concern for their welfare. | Scale: 14 items (e.g., “I often go out of my way to help another person.”).  [33]  Response option: 5-point scale (1 = extremely uncharacteristic, 5 = extremely characteristic).  Score: A mean of the sum of the items is calculated. |
| Individualism - Collectivism | Collectivism includes a sense of belonging and duty to in-groups, interdependence with group members, maintenance of one’s social status, seeking harmony and avoiding conflicts, and a preference for an indirect communication style. Individualism is primarily related to valuing personal independence, the distinction of the self from others, dominance on self-reliance, a striving for personal goals, self-interest and personal goals over those of society, and a preference for a direct communication style. | The Auckland Individualism and Collectivism Scale (AICS), defines three dimensions of individualism: (a) responsibility (acknowledging one’s responsibility for one’s actions), (b) uniqueness (distinction of the self from the other), and (c) competitiveness (striving for personal goals is one’s prime interest). The scale also defines two dimensions of collectivism: (a) advice (seeking advice from people close to one, before taking decisions) and (b) harmony (seeking to avoid conflict).  Scale: 26 items (e.g., “Winning is important to me.”)[34]  Response option: 6-point scale (1 = never, 6 = always).  Score: A mean of the sum of the items per facet is calculated. |
| Empathy  Toronto Empathy Questionnaire (TEQ) | Emotional empathy is commonly thought of as an emotional reaction (e.g., compassion) to another’s emotional response (e.g., sadness). This reaction is not dependent on a cognitive understanding of why a person is suffering [35], although it may facilitate understanding and action. | Scale: 16 items (e.g., “I get a strong urge to help when I see someone who is upset.”) [36]  Scale: 5-point scale (0 = never, 1 = rarely, 2 = sometimes, 3 = often, 4 = always).  Score: A mean of the sum of the items per facet is calculated. |
| **Explorative** |  |  |
| Big 5 | Taxonomy of personality. Assumes that personality is ordered hierarchically from a large number of specific traits to a much smaller number of general characteristics [14–16]  Hierarchical model that groups behavioral measures into higher-order clusters, consisting of Openness to experience, Conscientiousness, Extraversion, Agreeableness, and Neuroticism.  Explains individual differences [37]. | Scale: 10 items, 2 items for each personality dimension (e.g., “I see myself as someone who is reserved.”)[17]  Response option: 5-point scale (1 = disagree strongly, 5 = agree strongly).  Score: A mean of the sum of these responses per facet is calculated. |

1. Ajzen I. Constructing a Theory of Planned Behavior Questionnaire. 2006.

2. Askelson NM, Campo S, Lowe JB, Smith S, Dennis LK, Andsager J. Using the theory of planned behavior to predict mothers’ intentions to vaccinate their daughters against HPV. J Sch Nurs. 2010;26: 194–202.

3. Horne R, Weinman J, Hankins M. The beliefs about medicines questionnaire: The development and evaluation of a new method for assessing the cognitive representation of medication. Psychol Health. 1999;14: 1–24. doi:10.1080/08870449908407311

4. Bruder M, Haffke P, Neave N, Nouripanah N, Imhoff R. Measuring individual differences in generic beliefs in conspiracy theories across cultures: Conspiracy Mentality Questionnaire. Front Psychol. 2013;4: 225.

5. Weber E, Blais A-R, Betz N. A Domain-Specific Risk-Attitude Scale: Measuring Risk Perceptions and Risk Behaviors. J Behav Decis Mak. 2002;15. doi:10.1002/bdm.414

6. Johnson JG, Wilke A, Weber EU. DOSPERT-G Bereichsspezifische Risikoskala – Deutsche Version Domain-specific Risk-taking Scale – German version. 2004.

7. Strathman A, Gleicher F, Boninger DS, Edwards CS. The consideration of future consequences: Weighing immediate and distant outcomes of behavior. J Pers Soc Psychol. 1994;66: 742.

8. Brewer NT, Chapman GB, Gibbons FX, Gerrard M, McCaul KD, Weinstein ND. Meta-analysis of the relationship between risk perception and health behavior: the example of vaccination. [Internet]. American Psychological Association; 2007. Available: http://psycnet.apa.org/journals/hea/26/2/136/

9. Fadda M, Galimberti E, RomanÃ^2^ L, Faccini M, Senatore S, Zanetti A, et al. Validation of a scale to measure parental psychological empowerment in the vaccination decision. J Public Health Res. 2017;6. doi:10.4081/jphr.2017.955

10. Bandura A. Social cognitive theory of self-regulation. Organ Behav Hum Decis Process. 1991;50: 248–287. doi:10.1016/0749-5978(91)90022-L

11. Schwarzer R, Jerusalem M. Generalized Self-Efficacy scale. Measures in health psychology: A user’s portfolio Causal and control beliefs. Windsor, UK: NFER-NELSON; pp. 35–37. Available: https://userpage.fu-berlin.de/health/engscal.htm

12. Fagerlin A, Zikmund-Fisher BJ, Ubel PA, Jankovic A, Derry HA, Smith DM. Measuring numeracy without a math test: development of the Subjective Numeracy Scale. Med Decis Mak Int J Soc Med Decis Mak. 2007;27: 672–680. doi:10.1177/0272989X07304449

13. Schwartz LM, Woloshin S, Black WC, Welch HG. The role of numeracy in understanding the benefit of screening mammography. Ann Intern Med. 1997;127: 966–972.

14. Digman JM. Higher-order factors of the Big Five. J Pers Soc Psychol. 1997;73: 1246–1256.

15. Kotov R, Gamez W, Schmidt F, Watson D. Linking “big” personality traits to anxiety, depressive, and substance use disorders: a meta-analysis. Psychol Bull. 2010;136: 768–821. doi:10.1037/a0020327

16. Markon KE, Krueger RF, Watson D. Delineating the Structure of Normal and Abnormal Personality: An Integrative Hierarchical Approach. J Pers Soc Psychol. 2005;88: 139–157. doi:10.1037/0022-3514.88.1.139

17. Rammstedt B, Kemper C, Klein M, Beierlein C, Kovaleva A. Eine kurze Skala zur Messung der fünf Dimensionen der Persönlichkeit: Big-Five-Inventory-10 (BFI-10) [A Short Scale for Assessing the Big Five Dimensions of Personality - 10 Item Big Five Inventory (BFI-10)]. Methoden – Daten – Anal. 2013;7: 233–249.

18. Paulhus D. Social desirable responding: The evolution of a construct. The role of constructs in psychological and education measurement. Mahwah, New Jersey: Earlbaum Publishers; 2002. pp. 49–69.

19. Kemper CJ, Beierlein C, Bensch D, Kovaleva A, Rammstedt B. Eine Kurzskala zur Erfassung des Gamma-Faktors sozial erwünschten Antwortverhaltens: die Kurzskala Soziale Erwünschtheit-Gamma (KSE-G). 2012;2012/25. Available: https://www.ssoar.info/ssoar/bitstream/handle/document/33958/ssoar-2012-kemper_et_al-Eine_Kurzskala_zur_Erfassung_des.pdf?sequence=1

20. Zingg A, Siegrist M. Measuring people’s knowledge about vaccination: developing a one-dimensional scale. Vaccine. 2012;30: 3771–3777.

21. Egede LE, Ellis C. Development and testing of the multidimensional trust in health care systems scale. J Gen Intern Med. 2008;23: 808.

22. Wiseman R, Watt C. Measuring superstitious belief: Why lucky charms matter. Personal Individ Differ. 2004;37: 1533–1541.

23. Betsch C. Präferenz für Intuition und Deliberation (PID). Z Für Differ Diagn Psychol. 2004;25: 179–197. doi:10.1024/0170-1789.25.4.179

24. Betsch C, Iannello P. Measuring individual differences in intuitive and deliberative decision making styles. A comparison of different measures. 2010; Available: https://publicatt.unicatt.it/handle/10807/33801

25. Kaplan GA, Goldberg DE, Everson SA, Cohen RD, Salonen R, Tuomilehto J, et al. Perceived health status and morbidity and mortality: evidence from the Kuopio ischaemic heart disease risk factor study. Int J Epidemiol. 1996;25: 259–265.

26. Hays RD, Morales LS. The RAND-36 measure of health-related quality of life. Ann Med. 2001;33: 350–357.

27. Lapsley DK, Hill PL. Subjective invulnerability, optimism bias and adjustment in emerging adulthood. J Youth Adolesc. 2010;39: 847–857.

28. Hill PL, Duggan PM, Lapsley DK. Subjective invulnerability, risk behavior, and adjustment in early adolescence. J Early Adolesc. 2012;32: 489–501.

29. Tangney JP, Baumeister RF, Boone AL. High Self-Control Predicts Good Adjustment, Less Pathology, Better Grades, and Interpersonal Success. J Pers. 2004;72: 271–324. doi:10.1111/j.0022-3506.2004.00263.x

30. Holm EJ, Holroyd K. The Daily Hassles Scale (Revised): Does it measure stress or symptoms? Behav Assess. 1992;14: 465–482. doi:10.1037/t57783-000

31. Kanner AD, Coyne JC, Schaefer C, Lazarus RS. Comparison of two modes of stress measurement: Daily hassles and uplifts versus major life events. J Behav Med. 1981;4: 1–39.

32. Cylus J, Papanicolas I. An analysis of perceived access to health care in Europe: How universal is universal coverage? Health Policy. 2015;119: 1133–1144.

33. Clark MS, Oullette R, Powell MC, Milberg S. Recipient’s mood, relationship type, and helping. J Pers Soc Psychol. 1987;53: 94.

34. Shulruf B, Hattie J, Dixon R. Intertwinement of individualist and collectivist attributes and response sets. J Soc Evol Cult Psychol. 2011;5: 51.

35. Rankin KP, Kramer JH, Miller BL. Patterns of cognitive and emotional empathy in frontotemporal lobar degeneration. Cogn Behav Neurol Off J Soc Behav Cogn Neurol. 2005;18: 28–36.

36. Spreng RN, McKinnon MC, Mar RA, Levine B. The Toronto Empathy Questionnaire: Scale development and initial validation of a factor-analytic solution to multiple empathy measures. J Pers Assess. 2009;91: 62–71.

37. van der Linden D, te Nijenhuis J, Bakker AB. The General Factor of Personality: A meta-analysis of Big Five intercorrelations and a criterion-related validity study. J Res Personal. 2010;44: 315–327. doi:10.1016/j.jrp.2010.03.003

38. Amin AB, Bednarczyk RA, Ray CE, Melchiori KJ, Graham J, Huntsinger JR, et al. Association of moral values with vaccine hesitancy. Nat Hum Behav. 2017;1: 873–880. doi:10.1038/s41562-017-0256-5

39. Gilkey MB, Reiter PL, Magnus BE, McRee A-L, Dempsey AF, Brewer NT. Validation of the vaccination confidence scale: a brief measure to identify parents at risk for refusing adolescent vaccines. Acad Pediatr. 2016;16: 42–49.
