# Supplementary material for: Beyond confidence: Development of a measure assessing the 5C psychological antecedents of vaccination
Source: PLoS One. 2018 Dec 7;13(12):e0208601. doi: 10.1371/journal.pone.0208601 (PMC6285469; doi:10.1371/journal.pone.0208601)
Supplement: S3 Table — (DOCX) [file pone.0208601.s003.docx]

**S3 Table**

| **Analysis 1** | | | | | **Analysis 2** | | | | **Analysis 3** | | | |
| --- | --- | --- | --- | --- | --- | --- | --- | --- | --- | --- | --- | --- |
|  | CONF | COMP | CONST | CALC | CONF | CONST | CALC | COMP | CONF | CONST | CALC | COMP |
| **CONF4** | 0.706 |  |  |  |  |  |  |  | 0.669 |  |  |  |
| **CONF1** | 0.786 |  |  |  | 0.801 |  |  |  | 0.773 |  |  |  |
| **CONF6** | 0.826 |  |  |  | 0.814 |  |  |  | 0.833 |  |  |  |
| **CONF7** | 0.851 |  |  |  | 0.875 |  |  |  | 0.867 |  |  |  |
| **CONV6** |  |  | 0.636 |  |  | 0.496 |  |  |  | 0.498 |  |  |
| **CONV2** |  |  | 0.531 |  |  | 0.58 |  |  |  | 0.584 |  |  |
| **CONV7** |  |  | 0.577 |  |  | 0.703 |  |  |  | 0.699 |  |  |
| **CONV8** |  | 0.336 | 0.649 |  |  | 0.833 |  |  |  | 0.832 |  |  |
| **CALC3** |  |  |  | 0.526 |  |  | 0.551 |  |  |  | 0.553 |  |
| **CALC9** |  |  |  | 0.728 |  |  | 0.754 |  |  |  | 0.754 |  |
| **CALC1** |  |  |  | 0.737 |  |  | 0.774 |  |  |  | 0.776 |  |
| **COMP5** |  | 0.382 |  |  |  |  |  |  |  |  |  | 0.412 |
| **COMP7** |  | 0.659 |  |  |  |  |  | 0.596 |  |  |  | 0.529 |
| **COMP4** | -0.342 | 0.493 |  |  | -0.319 |  |  | 0.489 |  |  |  | 0.594 |
| **COMP6** |  | 0.589 |  |  |  | 0.305 |  | 0.65 |  | 0.305 |  | 0.66 |
| CONF3_R | -0.774 |  |  |  | -0.754 |  |  |  |  |  |  |  |
| CONF8 | 0.729 |  |  |  |  |  |  |  |  |  |  |  |
| COMP8 | -0.682 | 0.313 |  |  |  |  |  |  |  |  |  |  |
| CONF5_R | -0.673 |  |  |  |  |  |  |  |  |  |  |  |
| CONV1_R | 0.662 |  |  |  |  |  |  |  |  |  |  |  |
| CALC8_R | 0.602 | -0.331 |  |  |  |  |  |  |  |  |  |  |
| COMP2_R | 0.579 |  |  |  |  |  |  |  |  |  |  |  |
| COMP9 | -0.524 |  |  |  |  |  |  |  |  |  |  |  |
| COMP1_R | 0.506 |  |  |  |  |  |  |  |  |  |  |  |
| COMP3 | -0.502 |  |  |  |  |  |  |  |  |  |  |  |
| CALC2 | -0.37 |  |  |  |  |  |  |  |  |  |  |  |
| CONF9 |  |  |  |  |  |  |  |  |  |  |  |  |
| COMP10_R |  | -0.505 |  |  |  |  |  | -0.524 |  |  |  |  |
| CONF2 |  | -0.446 |  |  |  |  |  |  |  |  |  |  |
| CONV3_R |  | -0.36 |  |  |  |  |  |  |  |  |  |  |
| CONV5_R |  |  |  |  |  |  |  |  |  |  |  |  |
| CALC10 |  |  |  |  |  |  |  |  |  |  |  |  |
| CONV9 |  |  | 0.53 |  |  |  |  |  |  |  |  |  |
| CONV4 |  |  | 0.409 |  |  |  |  |  |  |  |  |  |
| COMP11_R |  |  |  |  |  |  |  |  |  |  |  |  |
