# Supplementary material for: Beyond confidence: Development of a measure assessing the 5C psychological antecedents of vaccination
Source: PLoS One. 2018 Dec 7;13(12):e0208601. doi: 10.1371/journal.pone.0208601 (PMC6285469; doi:10.1371/journal.pone.0208601)
Supplement: S4 Table — This is an intermediate product and should not be used. This intermediate version is only provided for transparency reasons. Instruction: Please evaluate how much you disagree or agree with the following statements (1 = strongly disagree, 2 = moderately disagree, 3 = neutral, 4 = moderately agree, 5 = strongly agree). Scoring: calculate mean scores of each sub-scale. Items with (R) were reverse-coded. (DOCX) [file pone.0208601.s004.docx]

**S4 Table**

| Confidence CONF1 CONF4 CONF6 CONF7 |
| --- |
| Impfungen sind im Allgemeinen effektiv.  Ich vertraue darauf, dass staatliche Behörden Entscheidungen im besten Interesse für die Allgemeinheit treffen, was das Angebot von Impfungen anbelangt.  Ich habe Vertrauen in die Sicherheit von Routineimpfungen.  Sich impfen zu lassen ist eine gute Möglichkeit, um vor Krankheiten geschützt zu sein. |
| Complacency COMP6 COMP7 COMP4 COMP5 |
| Was impfen angeht bin ich vollkommen unentschieden.  Über das Thema Impfen habe ich noch nie so richtig nachgedacht.  Ich finde, dass genauso viel für das Impfen spricht wie dagegen.  Die Entscheidung mich impfen zu lassen treffe ich auf Grundlage meines Gefühls. |
| Constraints CONV2 CONV6 CONV7 CONV8 |
| Es ist für mich umständlich eine Impfung zu erhalten.  Mein Unwohlsein bei Arztbesuchen hält mich vom Impfen ab.  Alltagsstress hält mich davon ab, mich impfen zu lassen.  Der erforderliche Aufwand einer Impfung hält mich vom Impfen ab. |
| Calculation CALC1 CALC3 CALC9 |
| Wenn ich über Impfungen nachdenke, gewichte ich Vor- und Nachteile.  Ein volles Verständnis über die Thematik des Impfens ist mir wichtig, bevor ich eine Entscheidung in Bezug auf das Impfen treffe.  Wenn ich darüber nachdenke mich impfen zu lassen, wäge ich sorgfältig die Risiken und den Nutzen ab. |

*Note*: This is an intermediate product and should not be used. This intermediate version is only provided for transparency reasons. Instruction: Please evaluate how much you disagree or agree with the following statements (1 = strongly disagree, 2 = moderately disagree, 3 = neutral, 4 = moderately agree, 5 = strongly agree). Scoring: calculate mean scores of each sub-scale. Items with (R) were reverse-coded.
