# Supplementary material for: Beyond confidence: Development of a measure assessing the 5C psychological antecedents of vaccination
Source: PLoS One. 2018 Dec 7;13(12):e0208601. doi: 10.1371/journal.pone.0208601 (PMC6285469; doi:10.1371/journal.pone.0208601)
Supplement: S5 Table — α = Cronbach’s alpha; bold correlations were expected. ** p < .01; r = Spearman-Brown correlation to examine reliability of two item measures; † the two agreeableness items correlated at .07. Therefore, only the item “I trust others easily and believe in the good in man” was selected. 1 assessed in sample A (n = 1033), 2 assessed in sample B (n = 412). SocDes = social desirability. (DOCX) [file pone.0208601.s005.docx]

|  | α | **Confidence** | **Constraints** | **Complacency** | **Calculation** |
| --- | --- | --- | --- | --- | --- |
|  |  | α = .88 | α = .77 | α = .67 | α = .75 |
| Vaccine acceptance^1^ | n.a. | .375** | -.252** | -.154** | -.141** |
| Intention to get vaccinated^1^ | n.a. | .840** | -.121** | -.220** | -.334** |
| Attitude towards vaccination^1,2^ | .94 | **.822**** | -.214** | -.386** | -.248** |
| Knowledge about vaccination^1^ | n.a. | **.814**** | -.147** | -.406** | -.280** |
| Beliefs about medicine - benefits^2^ | .61 | **.325**** | -.026 | -.147** | -.006 |
| Beliefs about medicine - harms^2^ | .60 | **-.353**** | .121* | .360** | .097* |
| Conspiracy mentality^2^ | .75 | **-.229**** | .027 | .206** | .018 |
| Empowerment – others’ experience^1^ | *r* =.501 | -.276** | **.150**** | .318** | .219** |
| Empowerment – personal experience^1^ | *r* =.484 | -.149** | **.043** | .319** | .120** |
| Perceived behavioral control^2^ | *r* = .78 | -.032 | **-.208**** | -.155** | .003 |
| Self efficacy^2^ | .80 | .008 | **-.127**** | -.171** | .156** |
| Perceived risk of diseases^1^ | n.a. | .559** | -.148** | **-.253**** | **-.208**** |
| Attitude towards risk (risk seeking)^2^ | .63 | -.07 | .143** | **.149**** | **-.189**** |
| Consideration of future consequences^2^ | .82 | .099* | -.118* | **-.187**** | .182** |
| Normative beliefs^2^ | .87 | .504** | -.201** | **-.321**** | -.161** |
| Perceived descriptive norm^2^ | n.a. | .443** | -.314** | **-.358**** | -.101* |
| Perceived risk of vaccination^1^ | n.a. | -.855** | .106** | .326** | **.336**** |
| Numeracy^2^ | n.a. | .087 | -.032 | -.097 | -.015 |
| SocDes – emphasize positive qualities | 0.44 | .099* | -.091 | -.017 | .138** |
| SocDes – downplay negative qualities | 0.45 | .015 | .126* | .125* | -.07 |
| Extraversion^2^ | *r* = .67 | -.008 | -.134** | -.110* | .008 |
| Agreeableness †^2^ | n.a. | .025 | -.006 | .042 | -.031 |
| Neuroticism^2^ | *r* = .32 | -.018 | .142** | .187** | .002 |
| Openness for experience^2^ | *r* = .46 | -.098* | .01 | .012 | .150** |
| Conscientiousness^2^ | *r* = .32 | .026 | -.09 | -.133** | .208** |

**S5 Table**

*Note:* α = Cronbach’s alpha; bold correlations were expected. ** p < .01; *r* = Spearman-Brown correlation to examine reliability of two item measures; † the two agreeableness items correlated at .07. Therefore, only the item “I trust others easily and believe in the good in man” was selected. ^1^ assessed in sample A (*n* = 1033), ^2^ assessed in sample B (*n* = 412). SocDes = social desirability.

**Supplementary Table S6. Convergent and discriminant validity and comparison to existing measures (Study 2)**

|  | Parental Attitudes about Childhood Vaccinations (PACV) | PACV short scale [38] | Vaccination Confidence Scale (VCS) | VCS short scale (4 items sub-scale benefit,  [39]) |
| --- | --- | --- | --- | --- |
|  | α = .88  *n* = 253 | α = .77  *n* = 253 | α = .86  *n* = 1003 | α = .86  *n* = 1003 |
| Confidence | -.508 | -.500 | .766 | .766 |
| Constraints | .333 | .378 | -.346 | -.270 |
| Complacency | .601 | .647 | -.588 | -.531 |
| Calculation | .282 | .313 | -.154 | -.104 |
| Coll. Resp. | -.369 | -.379 | .398 | .352 |

*Note*: all *p*s < .001.

**Supplementary Figure S1. Means of existing hesitancy and confidence scales (Study 3).** For the means, each variable was transformed into POMP values [0,100] to allow for direct comparison of the mean values: percent of maximum possible [((observed - minimum)/(maximum - minimum)) x 100]. The PACV and the VAS were recoded for more convenient comparison. Higher values express more acceptance/positive attitudes/confidence.

**
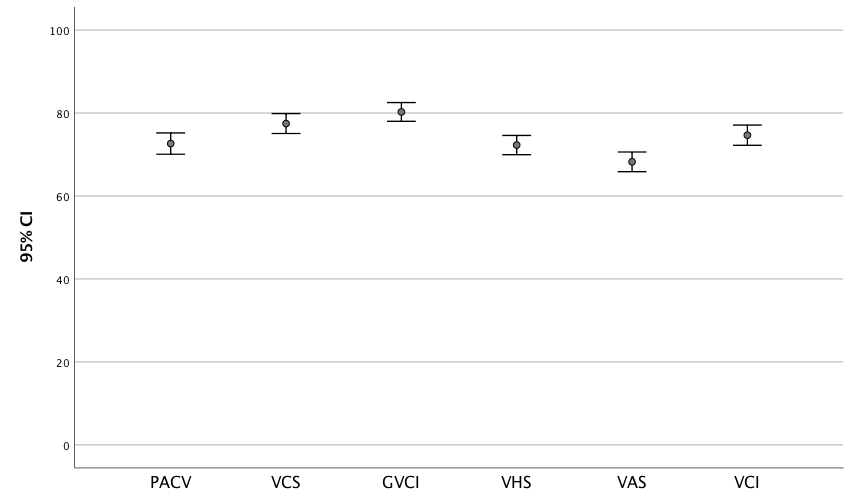
**

References

1. Ajzen I. Constructing a Theory of Planned Behavior Questionnaire. 2006.

2. Askelson NM, Campo S, Lowe JB, Smith S, Dennis LK, Andsager J. Using the theory of planned behavior to predict mothers’ intentions to vaccinate their daughters against HPV. J Sch Nurs. 2010;26: 194–202.

3. Horne R, Weinman J, Hankins M. The beliefs about medicines questionnaire: The development and evaluation of a new method for assessing the cognitive representation of medication. Psychol Health. 1999;14: 1–24. doi:10.1080/08870449908407311

4. Bruder M, Haffke P, Neave N, Nouripanah N, Imhoff R. Measuring individual differences in generic beliefs in conspiracy theories across cultures: Conspiracy Mentality Questionnaire. Front Psychol. 2013;4: 225.

5. Weber E, Blais A-R, Betz N. A Domain-Specific Risk-Attitude Scale: Measuring Risk Perceptions and Risk Behaviors. J Behav Decis Mak. 2002;15. doi:10.1002/bdm.414

6. Johnson JG, Wilke A, Weber EU. DOSPERT-G Bereichsspezifische Risikoskala – Deutsche Version Domain-specific Risk-taking Scale – German version. 2004.

7. Strathman A, Gleicher F, Boninger DS, Edwards CS. The consideration of future consequences: Weighing immediate and distant outcomes of behavior. J Pers Soc Psychol. 1994;66: 742.

8. Brewer NT, Chapman GB, Gibbons FX, Gerrard M, McCaul KD, Weinstein ND. Meta-analysis of the relationship between risk perception and health behavior: the example of vaccination. [Internet]. American Psychological Association; 2007. Available: http://psycnet.apa.org/journals/hea/26/2/136/

9. Fadda M, Galimberti E, RomanÃ^2^ L, Faccini M, Senatore S, Zanetti A, et al. Validation of a scale to measure parental psychological empowerment in the vaccination decision. J Public Health Res. 2017;6. doi:10.4081/jphr.2017.955

10. Bandura A. Social cognitive theory of self-regulation. Organ Behav Hum Decis Process. 1991;50: 248–287. doi:10.1016/0749-5978(91)90022-L

11. Schwarzer R, Jerusalem M. Generalized Self-Efficacy scale. Measures in health psychology: A user’s portfolio Causal and control beliefs. Windsor, UK: NFER-NELSON; pp. 35–37. Available: https://userpage.fu-berlin.de/health/engscal.htm

12. Fagerlin A, Zikmund-Fisher BJ, Ubel PA, Jankovic A, Derry HA, Smith DM. Measuring numeracy without a math test: development of the Subjective Numeracy Scale. Med Decis Mak Int J Soc Med Decis Mak. 2007;27: 672–680. doi:10.1177/0272989X07304449

13. Schwartz LM, Woloshin S, Black WC, Welch HG. The role of numeracy in understanding the benefit of screening mammography. Ann Intern Med. 1997;127: 966–972.

14. Digman JM. Higher-order factors of the Big Five. J Pers Soc Psychol. 1997;73: 1246–1256.

15. Kotov R, Gamez W, Schmidt F, Watson D. Linking “big” personality traits to anxiety, depressive, and substance use disorders: a meta-analysis. Psychol Bull. 2010;136: 768–821. doi:10.1037/a0020327

16. Markon KE, Krueger RF, Watson D. Delineating the Structure of Normal and Abnormal Personality: An Integrative Hierarchical Approach. J Pers Soc Psychol. 2005;88: 139–157. doi:10.1037/0022-3514.88.1.139

17. Rammstedt B, Kemper C, Klein M, Beierlein C, Kovaleva A. Eine kurze Skala zur Messung der fünf Dimensionen der Persönlichkeit: Big-Five-Inventory-10 (BFI-10) [A Short Scale for Assessing the Big Five Dimensions of Personality - 10 Item Big Five Inventory (BFI-10)]. Methoden – Daten – Anal. 2013;7: 233–249.

18. Paulhus D. Social desirable responding: The evolution of a construct. The role of constructs in psychological and education measurement. Mahwah, New Jersey: Earlbaum Publishers; 2002. pp. 49–69.

19. Kemper CJ, Beierlein C, Bensch D, Kovaleva A, Rammstedt B. Eine Kurzskala zur Erfassung des Gamma-Faktors sozial erwünschten Antwortverhaltens: die Kurzskala Soziale Erwünschtheit-Gamma (KSE-G). 2012;2012/25. Available: https://www.ssoar.info/ssoar/bitstream/handle/document/33958/ssoar-2012-kemper_et_al-Eine_Kurzskala_zur_Erfassung_des.pdf?sequence=1

20. Zingg A, Siegrist M. Measuring people’s knowledge about vaccination: developing a one-dimensional scale. Vaccine. 2012;30: 3771–3777.

21. Egede LE, Ellis C. Development and testing of the multidimensional trust in health care systems scale. J Gen Intern Med. 2008;23: 808.

22. Wiseman R, Watt C. Measuring superstitious belief: Why lucky charms matter. Personal Individ Differ. 2004;37: 1533–1541.

23. Betsch C. Präferenz für Intuition und Deliberation (PID). Z Für Differ Diagn Psychol. 2004;25: 179–197. doi:10.1024/0170-1789.25.4.179

24. Betsch C, Iannello P. Measuring individual differences in intuitive and deliberative decision making styles. A comparison of different measures. 2010; Available: https://publicatt.unicatt.it/handle/10807/33801

25. Kaplan GA, Goldberg DE, Everson SA, Cohen RD, Salonen R, Tuomilehto J, et al. Perceived health status and morbidity and mortality: evidence from the Kuopio ischaemic heart disease risk factor study. Int J Epidemiol. 1996;25: 259–265.

26. Hays RD, Morales LS. The RAND-36 measure of health-related quality of life. Ann Med. 2001;33: 350–357.

27. Lapsley DK, Hill PL. Subjective invulnerability, optimism bias and adjustment in emerging adulthood. J Youth Adolesc. 2010;39: 847–857.

28. Hill PL, Duggan PM, Lapsley DK. Subjective invulnerability, risk behavior, and adjustment in early adolescence. J Early Adolesc. 2012;32: 489–501.

29. Tangney JP, Baumeister RF, Boone AL. High Self-Control Predicts Good Adjustment, Less Pathology, Better Grades, and Interpersonal Success. J Pers. 2004;72: 271–324. doi:10.1111/j.0022-3506.2004.00263.x

30. Holm EJ, Holroyd K. The Daily Hassles Scale (Revised): Does it measure stress or symptoms? Behav Assess. 1992;14: 465–482. doi:10.1037/t57783-000

31. Kanner AD, Coyne JC, Schaefer C, Lazarus RS. Comparison of two modes of stress measurement: Daily hassles and uplifts versus major life events. J Behav Med. 1981;4: 1–39.

32. Cylus J, Papanicolas I. An analysis of perceived access to health care in Europe: How universal is universal coverage? Health Policy. 2015;119: 1133–1144.

33. Clark MS, Oullette R, Powell MC, Milberg S. Recipient’s mood, relationship type, and helping. J Pers Soc Psychol. 1987;53: 94.

34. Shulruf B, Hattie J, Dixon R. Intertwinement of individualist and collectivist attributes and response sets. J Soc Evol Cult Psychol. 2011;5: 51.

35. Rankin KP, Kramer JH, Miller BL. Patterns of cognitive and emotional empathy in frontotemporal lobar degeneration. Cogn Behav Neurol Off J Soc Behav Cogn Neurol. 2005;18: 28–36.

36. Spreng RN, McKinnon MC, Mar RA, Levine B. The Toronto Empathy Questionnaire: Scale development and initial validation of a factor-analytic solution to multiple empathy measures. J Pers Assess. 2009;91: 62–71.

37. van der Linden D, te Nijenhuis J, Bakker AB. The General Factor of Personality: A meta-analysis of Big Five intercorrelations and a criterion-related validity study. J Res Personal. 2010;44: 315–327. doi:10.1016/j.jrp.2010.03.003

38. Amin AB, Bednarczyk RA, Ray CE, Melchiori KJ, Graham J, Huntsinger JR, et al. Association of moral values with vaccine hesitancy. Nat Hum Behav. 2017;1: 873–880. doi:10.1038/s41562-017-0256-5

39. Gilkey MB, Reiter PL, Magnus BE, McRee A-L, Dempsey AF, Brewer NT. Validation of the vaccination confidence scale: a brief measure to identify parents at risk for refusing adolescent vaccines. Acad Pediatr. 2016;16: 42–49.
