# Supplementary material for: Beyond confidence: Development of a measure assessing the 5C psychological antecedents of vaccination
Source: PLoS One. 2018 Dec 7;13(12):e0208601. doi: 10.1371/journal.pone.0208601 (PMC6285469; doi:10.1371/journal.pone.0208601)
Supplement: S6 Table — All ps < .001. (DOCX) [file pone.0208601.s006.docx]

**S6 Table**

|  | Parental Attitudes about Childhood Vaccinations (PACV) | PACV short scale [1] | Vaccination Confidence Scale (VCS) | VCS short scale (4 items sub-scale benefit,  [2]) |
| --- | --- | --- | --- | --- |
|  | α = .88  *n* = 253 | α = .77  *n* = 253 | α = .86  *n* = 1003 | α = .86  *n* = 1003 |
| Confidence | -.508 | -.500 | .766 | .766 |
| Constraints | .333 | .378 | -.346 | -.270 |
| Complacency | .601 | .647 | -.588 | -.531 |
| Calculation | .282 | .313 | -.154 | -.104 |
| Coll. Resp. | -.369 | -.379 | .398 | .352 |

*Note*: all *p*s < .001.

1. Amin AB, Bednarczyk RA, Ray CE, Melchiori KJ, Graham J, Huntsinger JR, et al. Association of moral values with vaccine hesitancy. Nat Hum Behav. 2017;1: 873–880. doi:10.1038/s41562-017-0256-5

2. Gilkey MB, Reiter PL, Magnus BE, McRee A-L, Dempsey AF, Brewer NT. Validation of the vaccination confidence scale: a brief measure to identify parents at risk for refusing adolescent vaccines. Acad Pediatr. 2016;16: 42–49.
