# Supplementary figures and images for: Beyond confidence: Development of a measure assessing the 5C psychological antecedents of vaccination
Source: PLoS One. 2018 Dec 7;13(12):e0208601. doi: 10.1371/journal.pone.0208601 (PMC6285469; doi:10.1371/journal.pone.0208601)

**S1 Fig**

**
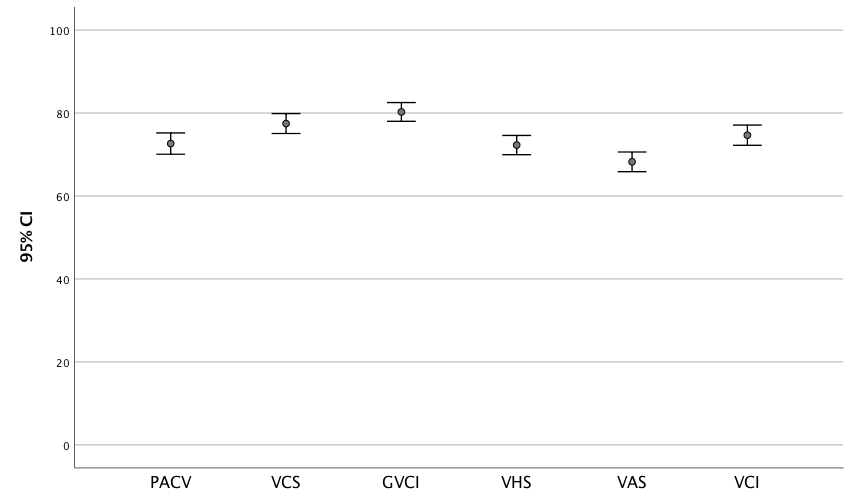
**

Supplement: S1 Fig — For the means, each variable was transformed into POMP values [0,100] to allow for direct comparison of the mean values: percent of maximum possible [((observed—minimum)/(maximum—minimum)) x 100]. The PACV and the VAS were recoded for more convenient comparison. Higher values express more acceptance/positive attitudes/confidence. (DOCX) [file pone.0208601.s007.docx]
